# Genes A27L and F13L as Genetic Markers for the Isolation of Recombinant Vaccinia Virus

**DOI:** 10.1038/s41598-019-52053-4

**Published:** 2019-10-30

**Authors:** María M. Lorenzo, Juana M. Sánchez-Puig, Rafael Blasco

**Affiliations:** Departamento de Biotecnología, Instituto Nacional de Investigación y Tecnología Agraria y Alimentaria (I.N.I.A.), Ctra. La Coruña km 7.5, E-28040 Madrid, Spain

**Keywords:** Viral vectors, Pox virus

## Abstract

After assembly in the cytosol, some Vaccinia virus particles go through a complex process that leads to virus egress and eventually cell-to-cell transmission. Intracellular particles are fully infectious, and therefore virus mutants lacking essential functions in the exit pathway are unable to form plaques but can multiply intracellularly. We isolated virus mutants in which two of the genes required for virus spread (F13L and A27L) were deleted independently or concurrently. The phenotypes of the mutant viruses were consistent with the need of A27L and F13L for intercellular virus transmission, the effect of the ΔA27L mutation being more severe than that of ΔF13L. Despite their defect in spread, ΔA27L mutant viruses could be expanded by infecting cell cultures at high multiplicity of infection, followed by the release of virions from infected cells by physical means. We developed a novel system for the isolation of recombinant Vaccinia virus in which selection is efficiently achieved by recovering plaque formation capacity after re-introduction of A27L into a ΔA27L virus. This system allowed the insertion of foreign DNA into the viral genome without the use of additional genetic markers. Furthermore, starting with a double mutant (ΔA27L-ΔF13L) virus, A27L selection was used in conjunction with F13L selection to mediate simultaneous dual insertions in the viral genome. This selection system facilitates combined expression of multiple foreign proteins from a single recombinant virus.

## Introduction

Vaccinia virus (VV), the best characterized member of the Poxviridae family, has been extensively used as an expression vector. Additionally, there is intense interest for its use as the backbone for recombinant vaccines, and as an oncolytic agent. Genetic modifications are usually introduced in the viral genome before use, with the exception of its original application as the smallpox vaccine and early oncolytic trials. In addition, some applications require the construction of multiple modifications of the viral genome or co-expression of several proteins^1–4^. Therefore, improvements of the methods to modify the viral genome are of interest.

Since the introduction of the first methods to insert genes in the VV genome^5,6^, the standard technique to obtain recombinant Poxviruses relies on homologous recombination between transfected plasmids and the viral genome replicating in the cytoplasm. This infection/transfection procedure generates only a small fraction of recombinant virus in the progeny population. In this regard, selective markers are commonly used to remove parental virus from the mixture to facilitate the isolation of recombinant virus^7^. One of the selection systems for VV is based on plaque size^8^, and it involves the use of the F13L gene as a selective genetic marker. The F13L gene is required for virus envelopment and therefore F13L-deficiency leads to very low virus propagation, resulting in a tiny-plaque phenotype^9,10^. Thus, re-introduction of the F13L gene in a F13L-deleted virus, with the concomitant plaque recovery, can be used as a genetic selection method.

The VV A27L gene encodes a 14 kDa protein that participates in virus attachment^11^, membrane fusion^12,13^, intracellular virus transport^14^ and the formation of enveloped virions^15^. Given the critical role for A27L in these processes, it is not surprising that inactivation or certain modifications of the A27L gene result in viruses with severely diminished plaque sizes^16,17^.

To expand the applications of the plaque-based VV selection system, here we explored the use of A27L as a selectable genetic marker, and developed a virus/plasmid pair to mediate the insertion of foreign DNA into the VV genome. We demonstrate that the ensuing system is reliable and highly efficient for the isolation of recombinant VV expressing foreign proteins. Furthermore, we show that A27L selection can be used in conjunction with the F13L system to simultaneously drive dual gene insertion into the viral genome.

## Results

### Isolation of v-ΔA27

**Figure 1:**
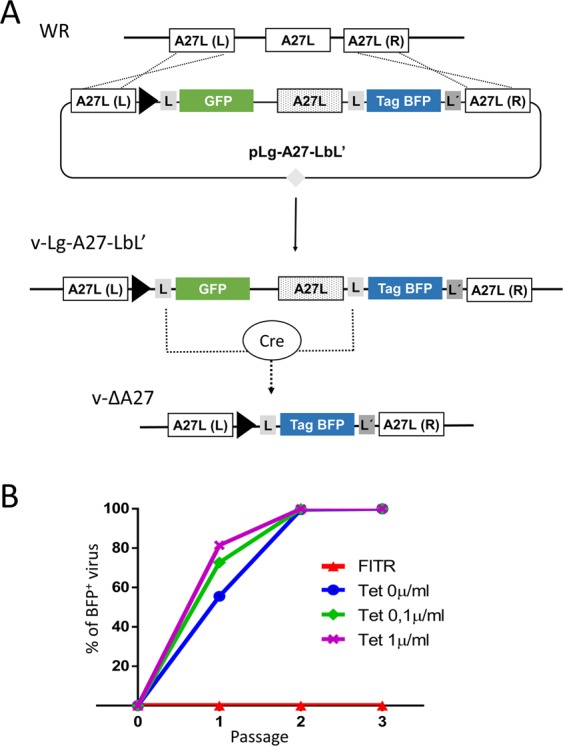
Isolation of v-∆A27. (**A**) Design of the deletion process. First, the normal A27L viral gene is replaced with a cassette containing GFP, a modified version of A27L (for synonymous codons, dotted box) and TagBFP. The position of the A27L recombination flanks A27L(L) and A27L(R), promoters (filled triangles) and LoxP (L) and Lox2272 (L′) sites is indicated. Note that GFP (but not TagBFP) is under the control of a Poxviral promoter. Virus v-Lg-A27-LbL′ was isolated after recombination of plasmid pLg-A27-LbL′ with the Vaccinia WR genome. Virus v-ΔA27L was then isolated through Cre-mediated recombination between LoxP (L) sequences. (**B**) Accumulation of BFP^+^ virus during passages in Cre-expressing cells. The percentage of BFP^+^ virus over total virus after serial passages is shown. Passage 0 corresponds to the initial virus population. The concentration of tetracycline used to induce Cre expression in the 293-Cre cell line is indicated. FITR corresponds to the parental 293 cell line not expressing Cre.

To explore the use the A27L gene in a selection system, we first isolated a VV defective in this gene. Due to the defect in cell-to-cell spread, it could be anticipated that the isolation of an A27L-deficient mutant (v-ΔA27) would be cumbersome. Therefore, to facilitate this procedure, we devised a strategy (outlined in Fig. 1A) to achieve A27L deletion in a manner that is independent of plaque isolation. First, the wild-type A27L gene was replaced with a LoxP-flanked (“floxed”) version of A27L, which was modified using synonymous codon substitutions to prevent homologous recombination with the normal A27L gene (Supplementary Fig. S1). To mediate the deletion of the A27L gene, we intended to use Cre-specific recombinase, which is highly effective in VV^18^. To allow direct monitoring of the deletion process, the TagGFP2 and TagBFP genes (which are derived from distinct fluorescent proteins and do not share significant homology) were placed at both sides of the A27L gene. A synthetic promoter was only included upstream of the GFP gene, in such a way that recombination between the LoxP sites can be detected as a switch from green to blue fluorescence (Fig. 1A).

Following the outlined strategy, the GFP/floxed-A27/BFP segment was inserted into the viral genome in place of the genomic A27L gene. The resulting virus, named vLg-A27-LbL′, was isolated by plaque purification and expanded. This virus displayed a normal plaque phenotype (not shown), thereby indicating that the codon changes introduced into A27L did not disturb A27L function to a significant extent. In a second step, A27L gene deletion was accomplished by repeatedly passaging vLg-A27-LbL′ in a cell line expressing Cre recombinase under the control of a Tet-inducible promoter. The emergence of BFP^+^ virus reached over 99% of the virus population in the second passage, as monitored by fluorescence microscopy (Fig. 1B). The increase in the percentage of BFP^+^ virus was faster with increasing concentrations of Tet inducer, but occurred even with basal expression of Cre recombinase (Fig. 1B, Tet 0 µg/ml). As expected, the enrichment of BFP^+^ virus paralleled the accumulation of tiny plaques, as predicted for an A27L-deficient virus. These observations were consistent with the unidirectional excision of the floxed TagGFP2-A27L gene cassette by Cre recombinase. After the fourth passage, v-ΔA27 was isolated by a limiting dilution step, which served to remove any remaining GFP^+^ virus from the passaged viral stock.

Using a similar procedure, a double F13L-A27L deletion mutant virus (v-ΔA27-ΔF13) was isolated, starting from virus mutant vRB12, which contains a deletion of the F13L gene^19^.

**Figure 2:**
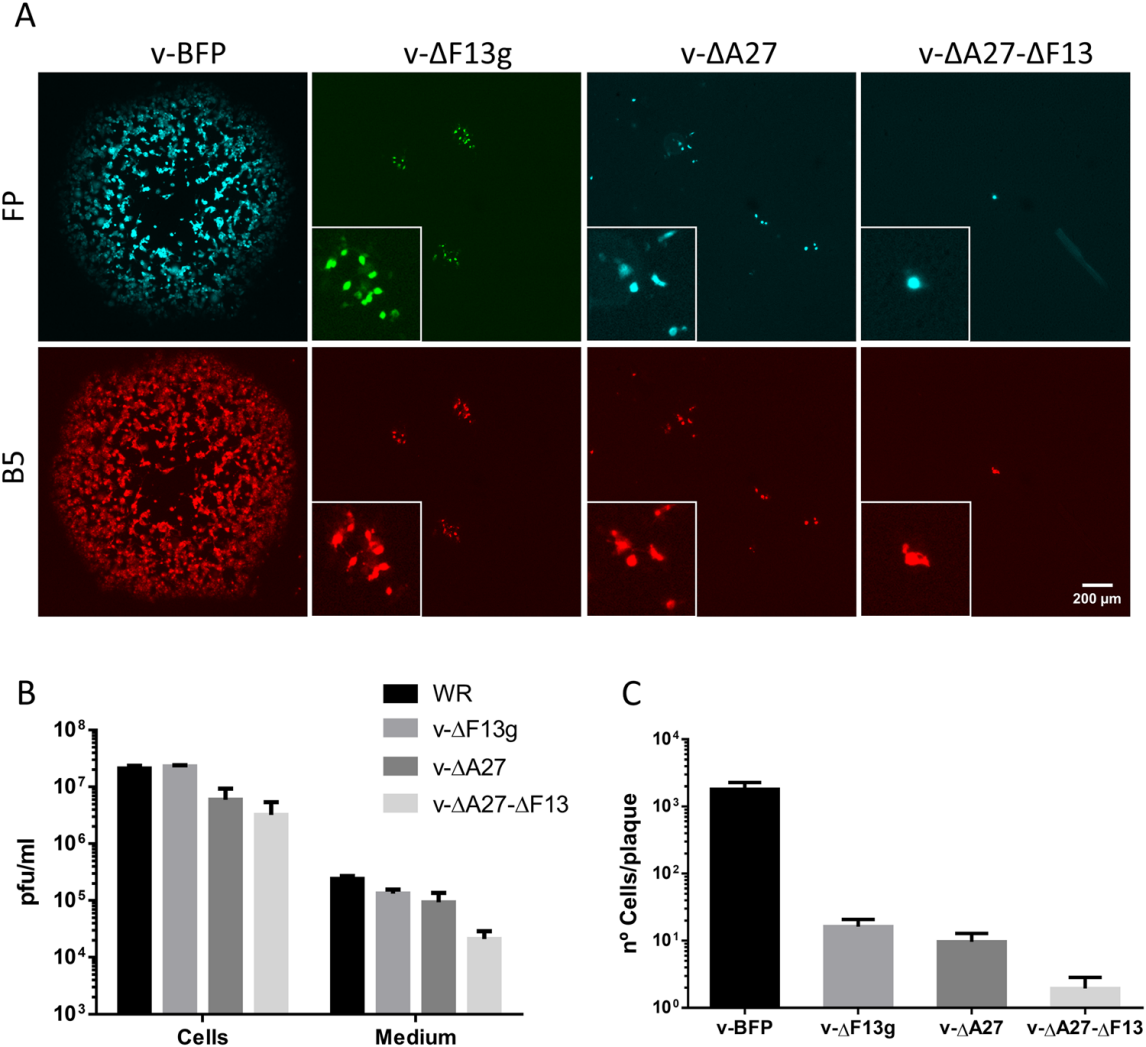
Characterization of A27-deficient viruses. (**A**) Plaque formation. Representative 3-day plaques of the indicated viruses as detected by the expression of fluorescent proteins (FP, top row) or by staining of infected cells with antibody against viral protein B5 (bottom row). Insets show enlargement of tiny plaques. (**B**) Production of extracellular and cell-associated virus. BSC-1 cells were infected at an MOI of 3 pfu/cell in triplicate. At 36 h post infection, virus in the cell culture medium and associated with the cells was titrated by plaque assay on fresh BSC-1 monolayers. (**C**) Plaque size. BSC-1 cells were infected with the viruses indicated, and the plaques were photographed after 72 h under the microscope. The number of fluorescent cells in the plaques (n = 35) were counted. Error bars represent standard deviation.

Initial characterization of v-ΔA27 confirmed the relevance of A27 protein for the intercellular transmission of viral infection. In agreement with previous reports^16^, the virus plaques produced by v-ΔA27 were tiny, even after prolonged incubation times. Indeed, they were slightly smaller than those of v-ΔF13L (Fig. 2A). Of note, v-ΔA27-ΔF13 produced even smaller plaques, which typically comprised 1 or 2 cells after 3 days, thereby indicating an almost complete absence of virus spread. Likewise, extracellular virus production showed an accumulative effect for the F13L and A27L deletions (Fig. 2B), reducing titres to 56%, 38% and 9% of those of the parental virus for the F13, A27 and F13/A27 deletions respectively.

In addition to the effects directly attributable to blockage of extracellular enveloped virus formation, we noted a slight but reproducible decrease in cell-associated virus titres as a result of the A27L deletion. This effect, which may be related to the involvement of A27L in the intracellular dispersal of virus from factories^14^, or to additional roles of the protein, was not pursued further.

### A27L-VV selection system

**Figure 3:**
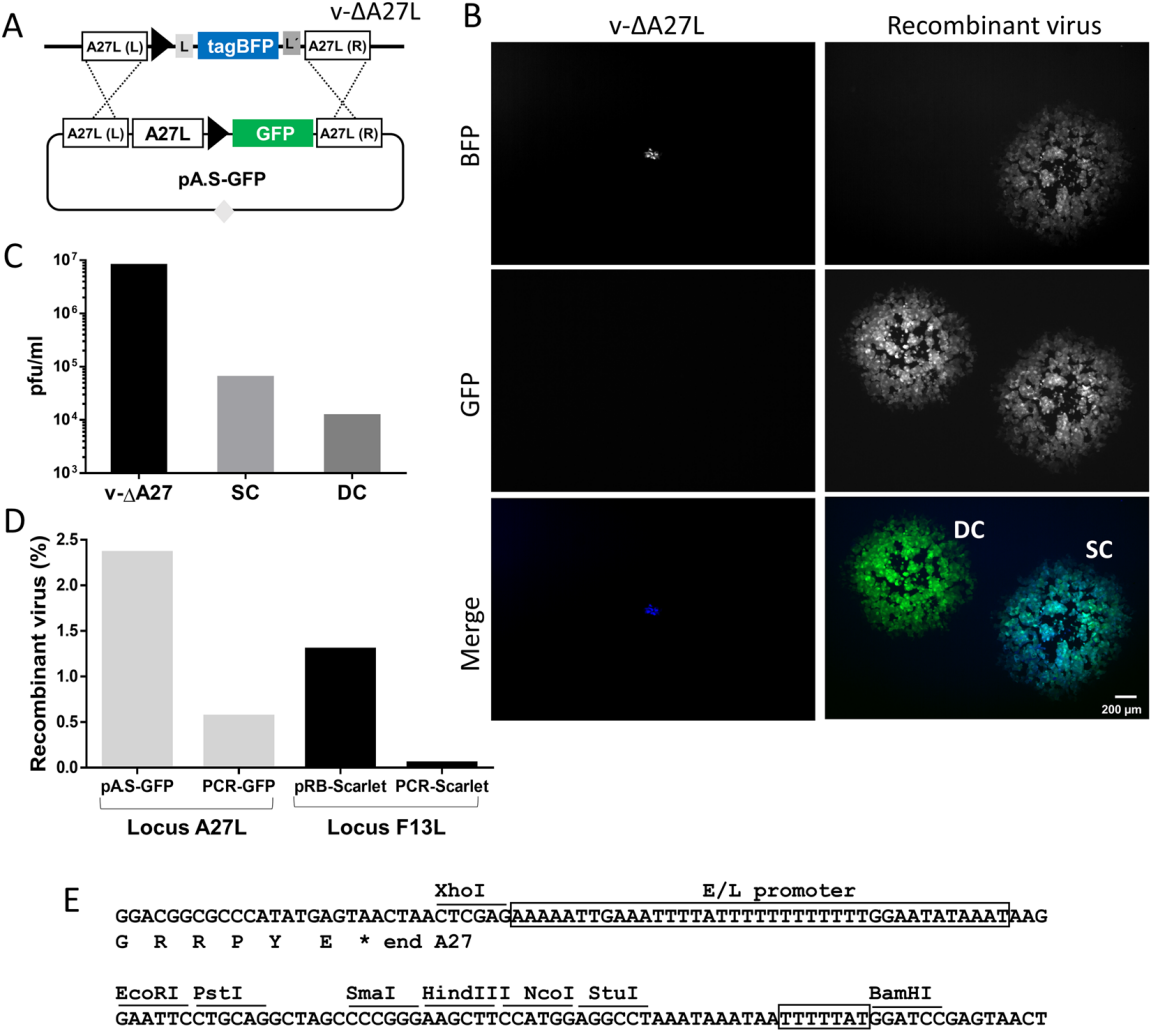
A27 selection system. (**A**) Schematic representation of the recombination event. The position of recombination flanks A27L (L) and A27L (R) is indicated. The black triangle indicates a poxviral synthetic early/late promoter. (**B**) Plaque phenotype of the different viruses produced at 2 days post-infection. In the left panels, the size of a representative plaque produced by the parental v-∆A27 is shown. Right panels show large plaques corresponding to the single cross-over (SC) and the double cross-over (DC) viruses. (**C**) Titration of the progeny from the infection/transfection. The titres for the parental (v-∆A27), SC and DC viruses are shown. (**D**) Comparison between A27 and F13 selection. The percentage of recombinant viruses with respect to the total progeny virus is shown, both for circular plasmids (pA.S-GFP and pRB-Scarlet) and for linear molecules (PCR-GFP and PCR-Scarlet). (**E**) Sequence of the pA.S plasmid around the multiple cloning site, where the end of the A27L gene, the downstream synthetic early/late promoter (E/L promoter), and relevant cloning sites are shown. Note that the promoter/cloning sequence is identical to that of plasmid pRB21^8^. Genes containing an ATG start codon can be inserted using the EcoRI-PstI-SmaI-HindIII sites. In addition, the NcoI site includes an initiator codon that allows in-frame cloning in the downstream StuI site. Sequence TTTTTAT (box) is a poxviral early transcription terminator. Sequence of plasmids pA.S, pA.S-tagGFP2 can be found in the Supplementary Information.

The defective phenotype of v-ΔA27 suggested that restoration of the A27L gene might serve as the basis for an efficient genetic selection system. To test this notion, we designed a plasmid to mediate the insertion of foreign DNA downstream of the A27L gene (Fig. 3A). The transfer plasmid, named pA.S contained the following: (i) A27L recombination flanks (566 and 551 nucleotides in length); (ii) the normal A27L gene and promoter area; and (iii) a Poxviral synthetic early/late strong promoter. Multiple cloning restriction sites were also included to assist gene insertion (Fig. 3E). As a first example, we tested the performance of the system using GFP as the foreign gene. To this end, plasmid pA.S-TagGFP2 (Fig. 3A) was transfected into cells infected with v-ΔA27 to permit insertion into the genome by homologous recombination. Since the recipient virus expressed TagBFP, viruses derived from diverse recombination events (large GFP^+^ plaques for the double cross-over product and large GFP^+^/BFP^+^ plaques for the single cross-over product, see Fig. 3B) were easily distinguished from the parental virus (tiny BFP^+^ plaques). Analysis of the progeny virus obtained from the infection/transfection experiment indicated that recombination products were present at a frequency of about 10^−2^, and that the single cross-over recombinant viruses accounted for 84% of the large viral plaques. Plaque isolation allowed us to isolate the final, stable, double cross-over recombinant virus using established procedures^20^. The high frequency of recombinant virus may indicate a degree of enrichment in the transfected culture, due to the deficiency of the parental virus in spread.

Isolation of recombinant virus was also feasible after transfection of linear PCR products that included the recombination flanks. However, transfection of linear molecules resulted in a significant decrease in the recombinant virus titre with respect to the circular plasmid. Both with plasmids and with linear DNA, A27 selection outperformed the previously described F13 selection system (Fig. 3D).

Overall, the A27 cloning system demonstrated high efficiency, required no selective media or special cell lines, and allowed rapid enrichment of recombinant virus. Furthermore, it offered several advantages over similar current selection methods, including the small size of the A27L gene, the higher rate of recombinant virus obtained, and the smaller plaque phenotype of the parental virus. In addition to these benefits, direct monitoring of the double/single cross-over nature of the viruses forming large plaques was straightforward due to the presence of the BFP gene in the parental virus.

### Combined use of VV A27L and F13L selection to achieve dual insertion

**Figure 4:**
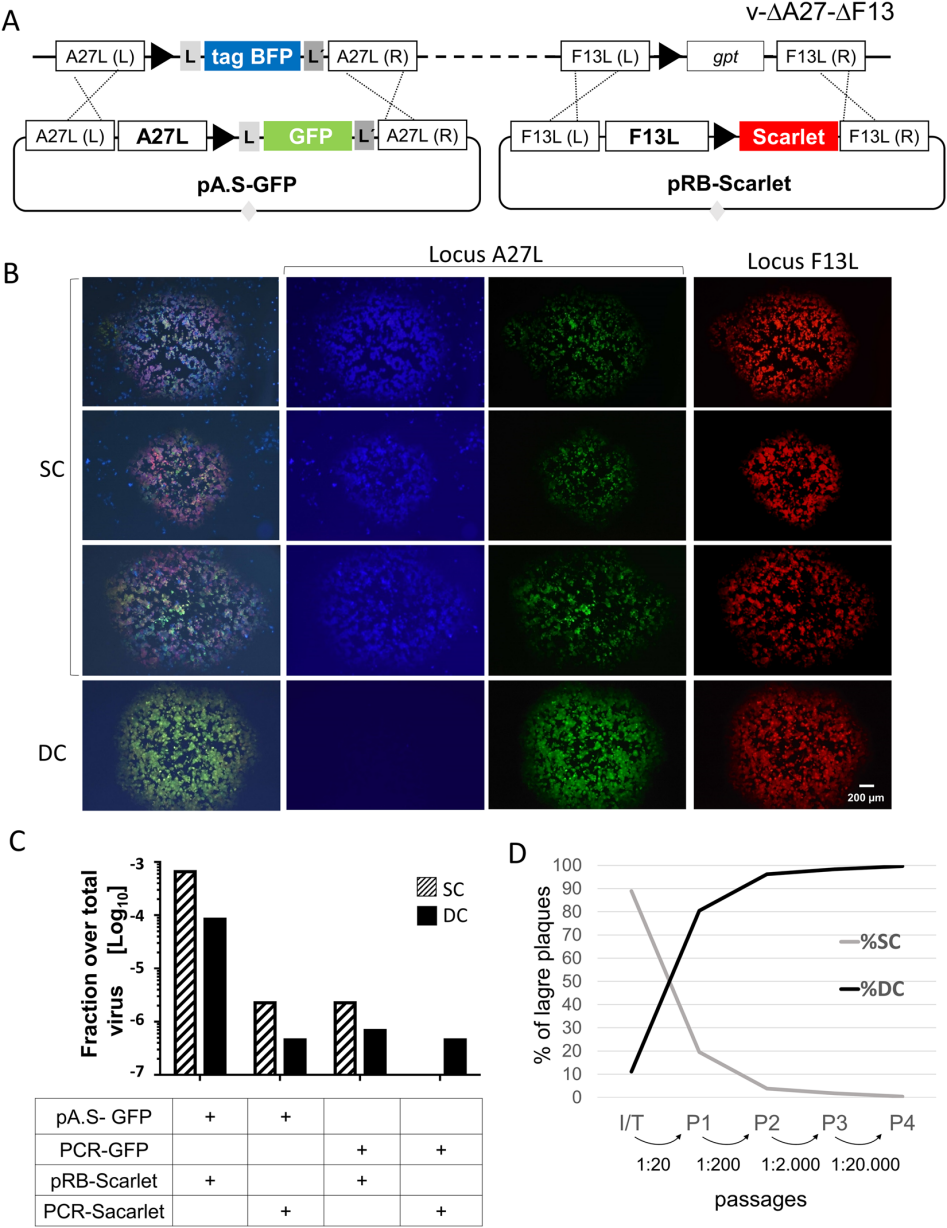
Testing of a double insertion in A27L and F13L loci. (**A**) Schematic representation of the virus v-∆A27-∆F13 and the plasmids used. Note that the *Escherichia coli* guanine phosphoribosyl transferase (*gpt*) gene substitutes for F13L gene in the parental virus, which is derived from vRB12^8^. (**B**) Plaque phenotypes for recombinant large-plaque viruses. Photographs of four distinct large plaques are shown. From left to right, images show RGB combined fluorescence, and blue, green, and red fluorescence. Viruses are denoted as SC (simple cross-over virus) and DC (double cross-over virus) with respect to the A27 locus, where the blue/green colour allows discrimination. (**C**) Comparison of circular versus linear molecules for transfection. The fraction of recombinant virus over total virus obtained from the infection/transfection step is shown for different plasmid/PCR combinations. (**D**) Enrichment of recombinant DC virus by serial passages. The virus from the infection/transfection was passaged in BSC-1 cells four consecutive times (P1–P4) using the fraction of the previous culture indicated for each passage. After each passage, the titre of SC and DC viruses (with respect to the A27L locus insertion) was determined by plaque assay.

A27L and F13L selection rely on the restoration of normal plaque-forming virus starting from a virus defective in plaque formation. Since we isolated a double ΔA27L/ΔF13L mutant and both genes are required to recover viral spread, we tested whether the same selection method could be used to simultaneously produce two insertion events. To this end, plasmids for recombination into the A27L and F13L loci were mixed and transfected into cells infected with v-ΔA27-ΔF13. To facilitate the monitoring of recombinant viruses, we included a GFP gene and the mScarlet gene, the latter encoding a red fluorescent protein derived from mCherry^21^. However, as the plasmids pA.S-Scarlet and pRB-TagGFP2 are both derived from pGEM plasmid, they share significant backbone homology, which may result in unwanted recombination events. Taking this into account, we performed parallel transfections in which linear PCR fragments lacking the plasmid vector sequences were used instead of complete plasmids. After transfection of plasmids, viruses forming large-plaques were detected in the viral progeny, indicating successful insertion of the A27L and F13L genes. Large plaques displayed a diversity of colours under the fluorescence microscope (Fig. 4B), indicating the expected single- or double- cross-over events in the two loci involved and the ensuing resolution of the unstable viruses by intra-molecular recombination. As before, recombination of linear PCR fragments was much less efficient than that of circular plasmids. Of note, large plaques displaying BFP fluorescence were obtained after transfection of a PCR product directed to the A27L locus. These viruses were not characterized, but presumably resulted from non-homologous recombination in the VV genome. Taking into account these results, we recommend using circular molecules for insertion.

The efficiency of the double insertion was significantly lower than that of the single insertion in the A27L locus. Indeed, the fraction of large plaques in the total virus progeny was reduced 10–50 fold with respect to that of the single gene setups. Since the low amount of recombinant virus hindered direct plaque isolation, we looked for an alternative strategy. Thus, to enrich in recombinant virus and to allow resolution of the single cross-over intermediates, we carried out serial passages at low multiplicity of infection (MOI), and followed the progression of fluorescent plaques by microscopy (Fig. 4D). As expected, the fraction of the stable recombinants increased with the passages and soon constituted the overwhelming majority of the viral stock. This experiment demonstrated that double insertion into the viral genome can achieve recombinant isolation using virus spread as the only genetic selection method.

#### Double coloured virus to facilitate plaque identification

**Figure 5:**
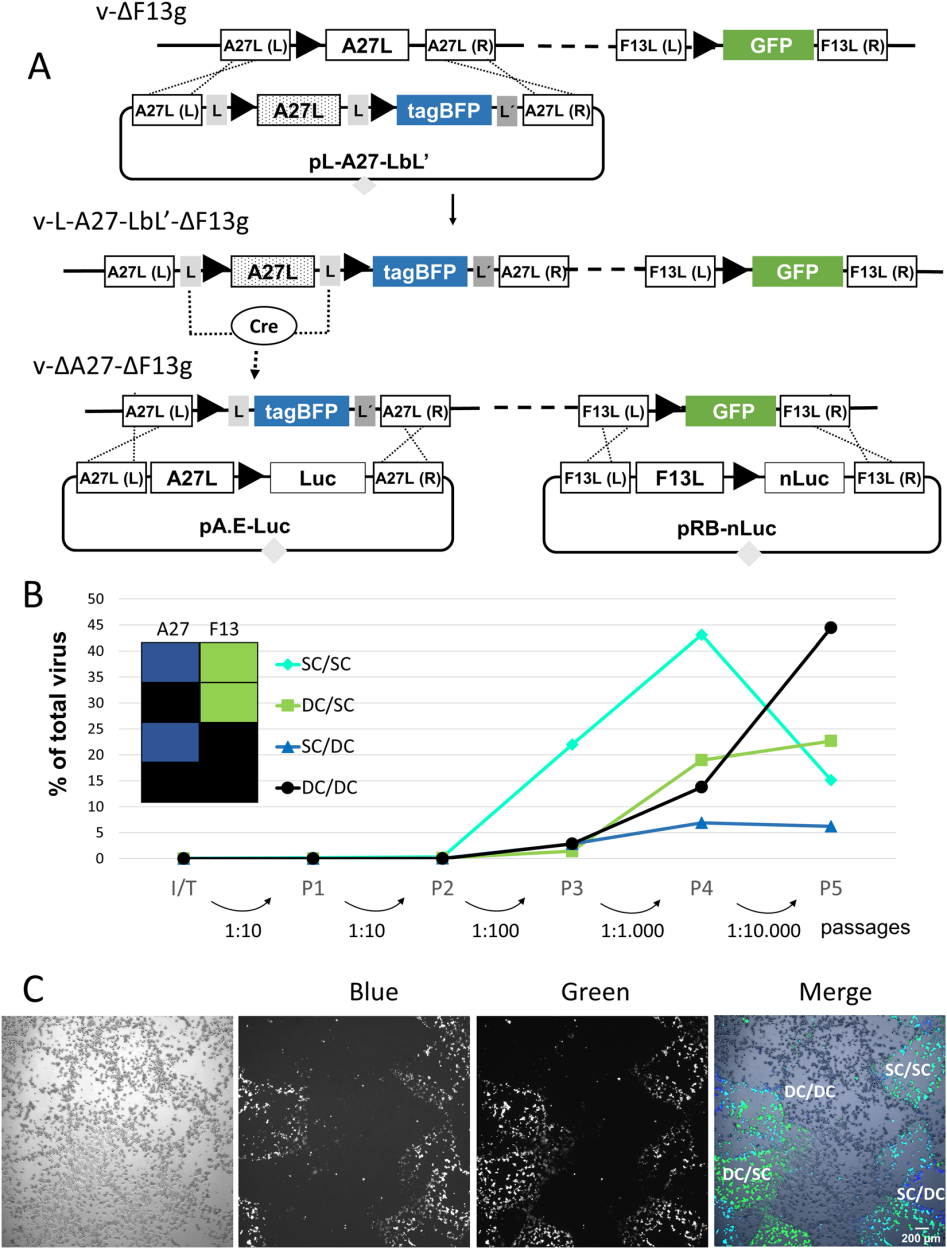
Double A27/F13 insertion system. (**A**) Isolation of v-∆A27-∆F13g and use in the selection system. The A27L gene was deleted using Cre-mediated recombination. Genetic elements are denoted as in Fig. 1. Below, design for the double insertion of luciferase genes. (**B**) Enrichment in recombinant viruses. The graph shows the enrichment in recombinant viruses along five consecutive passages (P1–P5) in which the fraction of the culture used to infect the following round is indicated. The fluorescence of the different large plaque-forming viruses is indicated in the inset. SC/DC and DC/SC indicate the single cross-over viruses for A27L and F13L insertions, respectively. Note that the final, stable, double cross-over for the two loci involved (DC/DC) has lost both blue and green fluorescence. (**C**) Images of the plaques of the fifth passage, where distinct recombinant viruses can be seen. Images were obtained with transmitted light (left), blue and green fluorescence. DC/DC non-fluorescent plaques correspond to the final recombinant virus.

To simplify identification of the progeny viruses, in the previous experiment we isolated virus recombinants expressing fluorescent proteins. However, to make the system more amenable for the expression of other foreign genes, we constructed an additional virus, termed v-ΔA27-ΔF13g, in which we included genes for TagBFP and TagGFP2 in place of the A27L and F13L genes, respectively (Fig. 5A). In this approach, double cross-over events are accompanied by the loss of both fluorescent proteins, and therefore the final recombinants are recognized as large plaques devoid of fluorescence. To test this system, we inserted the genes coding for firefly luciferase and nanoLuc into the A27L and F13L recombination plasmids, respectively. After mixed transfection of the two plasmids in v-ΔA27-ΔF13g-infected cells, the progeny virus was found to contain a low proportion of viruses with a large-plaque phenotype (approximately 10^−4^). Most large plaques displayed fluorescence, thereby hindering the direct isolation of the non-fluorescent double recombinant. From this mixed population, the double recombinant could be isolated by serial plaque isolation starting from a fluorescent single recombinant. As an alternative, we carried out serial passages of the initial virus population to enrich for A27L+ F13L+ viruses. We then determined the amount of different virus recombinants after each infection round (Fig. 5B). After four or five 1-day passages, large plaques accounted for a significant fraction of the virus population, thereby facilitating the isolation of the final recombinant (non-fluorescent plaques) by a standard plaque-picking procedure. To test the reliability of the system, we isolated ten large virus plaques using this procedure and confirmed that each of them were virus recombinants expressing both Luc and nanoLuc (not shown). This experiment showed the efficiency and consistency of the double selection system to isolate double recombinants.

## Discussion

Intercellular transmission of VV, and hence virus plaque formation, is dependent on the acquisition of the extracellular virus envelope, which contains several virally encoded proteins. Given that VV produces fully infectious virions in the cytosol, viruses impaired in spread can still be isolated and grown to high titres in the laboratory, as first exemplified by the isolation of a F13L-deficient virus^9,10^. However, isolation of viruses that do not form plaques is difficult, since both virus growth and visual identification are limited by the block in virus spread. Here, we describe an efficient method to isolate such viruses by using Cre recombinase to excise DNA from the viral genome^18^ in the absence of plaque isolation.

It has been reported that a vaccinia virus with the A27L gene repressed or deleted produces infectious cell-associated virus and small plaques, consistent with a deficiency in virus envelopment^14–16^. We have exploited this virus deficiency to improve the existing cloning vectors that use virus plaque formation to select virus recombinants. Of note, a point mutation in the A27L gene producing a small-plaque phenotype was previously proposed as an approach to select VV recombinants^22^.

The A27L selection system described here provides a novel insertion site in the vaccinia genome, can be used in unmodified cell lines, does not rely on the use of drugs or antibiotics, and renders marker-free recombinant viruses. In addition, the nature of the A27L mutation (deletion of all the A27L coding sequence) prevents the occurrence of large plaques as the result of spontaneous reversions. Although based on similar concepts, the A27L system offers several advantages over the classical F13L version. First, the A27L gene is smaller than F13L, and thus the plasmids for insertion in the A27L locus are easier to manipulate. Also, the efficiency of the A27L system was higher (see Fig. 3D), which may reflect the more profound phenotypic defect of the A27L-deficient virus. Finally, the inclusion of a fluorescent protein gene in place of the A27L gene facilitates the identification of the recombinant viruses generated during isolation.

The A27L/F13L system allows for straightforward insertion of two DNA segments in distant genomic loci. Notably, the only practical difference between the respective A27L and F13L selection protocols is the starting virus, since the same selection principle (i.e. plaque formation/virus cell-to-cell virus spread) is applied for the isolation of the double recombinant virus.

VV has considerable insertion capacity, thereby allowing the simultaneous expression of multiple genes. The availability of multiple insertion sites will facilitate the construction of complex recombinant viruses for various applications.

## Materials and Methods

### Cells

BSC-1 cells (ATCC CCL-26) were grown in Eagle’s Minimal Essential Medium (EMEM) supplemented with 0.1 mg/ml penicillin, 0.1 mg/ml streptomycin, 2 mM L-glutamine (Lonza) and 5% foetal bovine serum (FBS).

Flp-In T-Rex 293 cells (Invitrogen) were maintained in DMEM supplemented with 0.1 mg/ml penicillin, 0.1 mg/ml streptomycin, 2 mM L-glutamine (BioWhittaker), 7% foetal bovine serum (FBS), 300 µg/ml Zeocin and 10 µg/ml blasticidin.

The 293-Cre cell line, inducibly expressing Cre recombinase, was derived from Flp-In-293 cells by Flp recombinase-mediated insertion of plasmid pcDNA-FRT-Cre using the Flp-In T-Rex system (Invitrogen) following manufacturer’s instructions. 293-Cre cells were grown in DMEM supplemented with 7% foetal bovine serum (FBS) containing 0.1 mg/ml penicillin, 0.1 mg/ml streptomycin, 2 mM L-glutamine (BioWhittaker), 10 µg/ml blasticidin and 100 µg/ml hygromycin. Routinely, for induction of Cre expression, cells were incubated with medium containing 1 µg/ml tetracycline for 18–24 h before infection. After virus adsorption, incubation was continued in medium containing 2% FBS and 1 µg/ml tetracycline.

### Plasmids

#### pLg-A27-LbL′

A segment containing an A27L gene modified by synonymous substitutions and flanked by LoxP (ATAACTTCGTATA GCATACAT TATACGAAGTTAT) and Lox2272 (ATAACTTCGTATA GGATACTT TATACGAAGTTAT) sequences, in addition to ∼400 bp A27L recombination flanks, was synthesized by GenScript Corp and inserted into plasmid pUC57-simple, to generate pL-A27-L. A cassette comprising a synthetic early/late promoter, a LoxP sequence and the gene TagGFP2 sequence was obtained by digestion of pRB-E/L-LgL (ML, unpublished results) with XhoI and BamHI. This cassette was then inserted between SalI/BglII sites of pL-A27-L to generate pLg-A27-L. Finally, plasmid pLg-A27-LbL′ was obtained by inserting the TagBFP gene^23^ flanked by LoxP and Lox 2272 sites between the XhoI and HindIII sites of pLg-A27-L.

pRB-Scarlet was obtained by insertion of the gene coding for mScarlet^21^ obtained from plasmid pmScarlet-i_NES_C1, a gift from Dorus Gadella (Addgene plasmid #85062; http://n2t.net/addgene:85062; RRID:Addgene_85062) by PCR amplification with oligonucleotides Scarlet5′ (CCGGTCGAATTCATGGTGAGCAAGGGCGAG, EcoRI site underlined) and Scarlet3′ (GCAGAATTCGAAGCTTGAGCTCG, HindIII and EcoRI sites underlined). The PCR fragment containing the Scarlet coding sequence was digested with EcoRI and HindIII and inserted between the corresponding sites of pRB21, thus placing the mScarlet-I gene downstream of the synthetic early/late promoter in pRB21^8^. The resulting plasmid, pRB-mScarlet-i-NES, is referred to herein as pRB-Scarlet.

#### A27L insertion plasmids

A plasmid was designed to mediate the reinsertion of the A27L gene, together with foreign sequences, into the A27L locus. This plasmid (pA-LE-R_R), containing A27L recombination flanks (566 and 551 bp), the A27L gene and an early/late promoter^24,25^, was obtained by synthesis of a DNA fragment and subsequent cloning in plasmid pUC57 (done by Genscript Corp). Subsequently, pA-LE-Scarlet-NES was obtained by insertion of the gene coding for mScarlet-i from pRB-Scarlet into the EcoRI/BamHI sites of pA-LE-R_R (Sanchez-Puig *et al*., in preparation). Then, plasmid pA.S-TagGFP2 was obtained by substituting the promoter-Scarlet gene cassette by the synthetic E/L promoter-TagGFP2 gene cassette derived from pRB-TagGFP2 (Supplementary Info). Finally, plasmid pA.S was obtained by inserting the synthetic E/L promoter-multiple cloning site region from pRB21 between the XhoI and BamHI sites in pA.S-TagGFP2.

#### pRB-nLuc

The sequence for nanoLuc^26^ was synthesized by GenScript Corp and inserted between the EcoRI and HindIII sites in pRB21 plasmid^8^.

#### pA.E-Luc

The Firefly luciferase coding sequence was obtained from plasmid pTM1-Luc (unpublished) by NcoI and XhoI digestion and was cloned in the pTIH plasmid^27^ using the same sites to generate pTIH-Luc. Finally, pA.S-Luc was generated by inserting the luciferase coding sequence, obtained by EcoRI / BamHI digestion of pTIH-Luc, and insertion between the corresponding sites in pA.S plasmid.

#### 293-Cre cell line

A codon-optimized version of the Cre gene was derived from plasmid pDIRE^28^ (a gift from Rolf Zeller, Addgene plasmid #26745; http://n2t.net/addgene:26745; RRID:Addgene_26745) and inserted into pRB21 to generate pRB-Cre. Subsequently, a DNA segment containing the Cre gene was amplified by PCR with oligonucleotides Cre5′ HindIII Fw (5′-CCCAAGCTTACCATGTCCAACCTGCTGACTGTGCAC-3′) and HF3400 (5′ CGTTCTAAAGCTAGTGCTATATCTCCC-3′ corresponding to a sequence in the F13L right recombination flank of pRB21). This PCR fragment was digested with HindIII and inserted into the unique HindIII site of plasmid pcDNA5/FRT/TO (Invitrogen). The final plasmid construct was used to generate a 293T-derived cell line inducibly expressing Cre, following the instructions of the Flp-In T-Rex system (Invitrogen). The resulting inducible cell line was termed 293-Cre.

### Isolation of vaccinia virus mutants

#### Deletion of the VV F13L gene

The F13L gene was removed from plasmid pRB-rsGFP^29^ as follows: a PCR product corresponding to the F13L left recombination flank was amplified from VV DNA using oligonucleotides VV_LL_ (5′-GCATATGCATGCTTTGTTAAAATAGATA-3′, SphI site underlined) and VV_RR_ (5′-CATTTTGCTCGAGCAGGTACCGATGCAA-3′, XhoI site underlined). This PCR product was digested with SphI and XhoI and inserted between the corresponding sites in pRB-rsGFP to generate plasmid prsGFP. Since the SphI-XhoI fragment removed spans the left flank and the F13L gene, insertion of the left F13L flank resulted in the net removal of the F13L gene.

BSC-1 cells in 6-well plates were infected with VV strain WR at a MOI of 0.05 plaque forming units (pfu) per cell and subsequently transfected with 2 µg of prsGFP complexed with Fugene HD (Promega). Infection was allowed to proceed for 3 days, and the progeny virus was plated in BSC-1 cells. Large GFP(+) plaques (simple recombinants) were isolated twice by plaque picking. In an ensuing plaque assay, several tiny, green fluorescent plaques were isolated and expanded in BSC-1 monolayers in 24-well plates. A culture well devoid of large plaques was selected for further amplification. Finally, the virus -termed v-∆F13g was characterized by western blot using antibody against F13 and by positive rescue of the normal plaque phenotype following re-insertion of the F13L gene.

#### Deletion of VV A27L gene

BSC-1 cells in 6-well plates were infected with virus WR, vRB12^30^ or vΔF13g at a MOI of 1 pfu/cell and transfected with plasmid pLg-A27-LbL′ using FuGeneHD (Promega). After 2 days, cells were disrupted by three cycles of freeze-thawing, and the released virus was used to infect fresh BSC-1 cell monolayers. Virus plaques showing green fluorescence were selected and isolated by plaque picking. After five consecutive rounds of plaque isolation, virus was expanded and titrated. Viruses obtained by insertion of this construct in WR, vRB12 and vΔF13g were termed vLg-A27-LbL′, v∆F13-Lg-A27-LbL′ and v-L-A27-LbL′-∆F13g respectively, and contained an A27L gene flanked by LoxP recombination sites. To promote deletion of A27L by Cre-mediated recombination, these viruses were passaged in 293-Cre cells pre-incubated for 18 h with medium containing tetracycline. For serial passaging, viruses in cultures were recovered after 24 h of infection and used as the inoculum for the following passage. Finally, to eliminate any residual A27L(+) virus, BSC-1 cells were infected with serial dilutions of the last passage. After 72 h the wells were observed by fluorescence microscopy, and the mutated A27L-deficient virus was recovered from a dilution that showed blue fluorescence but was devoid of GFP fluorescence. The final virus stocks were amplified from these infected cultures and termed v-∆A27, v-∆A27-∆F13 or v-∆A27-∆F13g when derived from VV WR, vRB12 or vΔF13g, respectively.

### Generation of VV recombinants

Virus recombinants were isolated using standard protocols^20^ using circular (plasmids) or linear (PCR-generated) molecules. Linear DNA fragments including the desired recombination flanks were PCR-amplified from plasmids containing flanks for the A27L or F13L genomic loci. For amplification of A27L constructs, PCR was performed with oligonucleotides pALE FI Fw (5′-TCCGCGCACATTTCCCCGAAAAGTG-3′) and pALE FD Rv (5′-GCCAAGCTCGCTTGCACGCCTCTGC-3′). For amplification of F13L constructs, PCR was performed with oligonucleotides pRB FI Fw (5′-GACTCACTATAGGGCGAATTGGGCC-3′) and pRB FD Rv (5′-AGGTGACACTATAGAATACTCAAGC-3′).

To generate recombinant virus, the previously described protocol for F13L selection^7^ was followed. Briefly, BSC-1 cells in 6-well plates were infected at an MOI of 0.05 pfu/cell with a VV mutant deficient in plaque formation (viruses deleted in A27L or F13L). After a 1-h adsorption period, cells were transfected with DNA (2 µg of plasmid or 1 µg of PCR fragment) using FuGeneHD, following the manufacturer’s instructions. After 2–3 days, cells were harvested and recombinant viruses were isolated by at least three consecutive rounds of plaque purification. Alternatively, where indicated, serial passages were carried out in BSC-1 monolayers in T75 culture flasks.

### Immunofluorescence microscopy

Cells grown on 6-well plates were infected for 48 h with viruses expressing a fluorescent protein (vBFP, v-∆A27 and v-∆A27-∆F13 express TagBFP and v-∆F13g expresses GFP). After this time, cells were washed twice with PBS and fixed by the addition of ice-cold 4% paraformaldehyde for 12 min. All subsequent incubations were carried out at room temperature. Cells were permeabilised by a 15-min incubation in PBS containing 0.1% Triton X-100 and then treated with PBS containing 0.1 M glycine for 5 min. They were then incubated with primary antibodies diluted in PBS-20% FCS for 30 min, followed by incubation with secondary antibodies diluted 1:400 in PBS-20% FCS. The following antibodies were used: rat monoclonal antibody anti-B5 diluted 1:100; and anti-rat IgG-Alexa Fluor 594 (Invitrogen). Finally, cells were washed extensively with PBS, mounted with FluorSave reagent (Calbiochem), and observed by fluorescence microscopy.

## Supplementary information

Supplementary Information
