# Supplementary material for: Genes A27L and F13L as Genetic Markers for the Isolation of Recombinant Vaccinia Virus
Source: Sci Rep. 2019 Oct 30;9:15684. doi: 10.1038/s41598-019-52053-4 (PMC6821840; doi:10.1038/s41598-019-52053-4)
Supplement: Supplementary file 1 — Supplementary Information [file 41598_2019_52053_MOESM1_ESM.pdf]

Genes A27L and F13L as Genetic Markers for the Isolation of Recombinant Vaccinia Virus

María M. Lorenzo, Juana M. Sanchez-Puig and Rafael Blasco*

Departamento de Biotecnología,
Instituto Nacional de Investigación y Tecnología Agraria y Alimentaria (I.N.I.A.)
Ctra. La Coruña km 7.5
E-28040 Madrid. Spain

Supplementary Information

```
A27L          1 ATGGACGGAACTCTTTTCCCCGGAGATGACGATCTTGCAATTCCAGCAAC     50
                |||||.||.||..|.||.||.||.||.||.||.||.||.||.||.||.||
A27L(syn)     1 ATGGATGGCACATTGTTTCCTGGCGACGATGACCTGGCTATCCCTGCTAC     50

A27L         51 TGAATTTTTTTCTACAAAGGCTGCTAAAAAGCCAGAGGCTAAACGCGAAG    100
                .||.||.||....||.||.||.||.||.||.||.||.||.||..|.||.|
A27L(syn)    51 AGAGTTCTTCAGCACTAAAGCCGCCAAGAAACCTGAAGCAAAGAGAGAGG    100

A27L        101 CAATTGTTAAAGCCGATGAAGACGACAATGAGGAAACTCTCAAACAACGG    150
                |.||.||.||.||.||.||.||.||.||.||.||.||..|.||.||..|.
A27L(syn)   101 CTATCGTGAAGGCAGACGAGGATGATAACGAAGAGACATTGAAGCAGAGA    150

A27L        151 CTAACTAATTTGGAAAAAAAGATTACTAATGTAACAACAAAGTTTGAACA    200
                .|.||.||..|.||.||.||.||.||.||.||.||.||.||.||.||.||
A27L(syn)   151 TTGACAAACCTTGAGAAGAAAATCACAAACGTTACTACTAAATTCGAGCA    200

A27L        201 AATAGAAAAGTGTTGTAAACGCAACGATGAAGTTCTATTTAGGTTGGAAA    250
                .||.||.||.||.||.||..|.||.||.||.||..|.||..|..|.||.|
A27L(syn)   201 GATTGAGAAATGCTGCAAGAGAAATGACGAGGTGTTGTTCCGACTTGAGA    250

A27L        251 ATCACGCTGAAACTCTAAGAGCGGCTATGATATCTCTGGCTAAAAAGATT    300
                |.||.||.||.||..|..|.||.||.|||||...|||.||.||.||.||.
A27L(syn)   251 ACCATGCAGAGACATTGCGGGCTGCAATGATTAGTCTTGCCAAGAAAATC    300

                                              ***
A27L        301 GATGTTCAGACTGGACGGCGCCCATATGAGTAACTTAA    338
                ||.||.||.||.||..||.|.||.||.||.|||.||..
A27L(syn)   301 GACGTGCAAACAGGTAGGAGACCTTACGAATAAGTTTT    348
```

60.9% identity (212/348 nucleotides).

Supplementary Figure S1.- Sequence of the A27L coding sequence with synonymous codon substitutions A27L. The A27L coding sequence was modified with synonymous codon changes. The modified sequence A27(syn) is shown aligned with the unmodified sequence of the WR VVstrain. Start (ATG) and stop (TAA) are indicated in green and by asterisks, respectively.

Plasmid pRB21

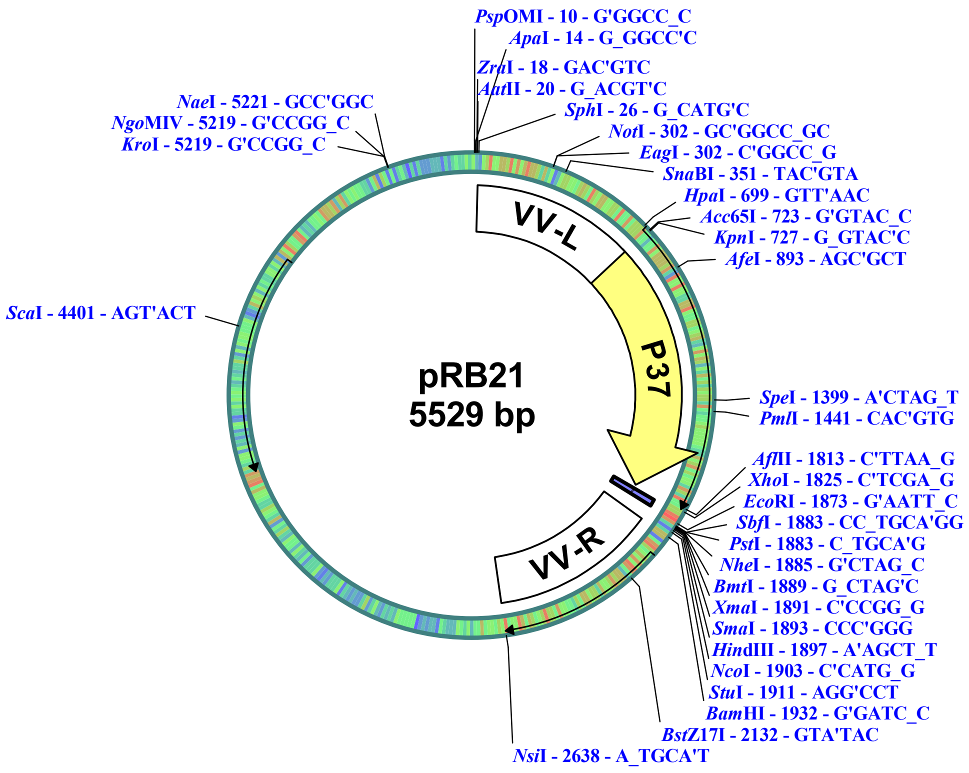


```
   1 GGGCGAATTG GGCCCGACGT CGCATGCGAT AAAGTTTCGA AACAGCAAAA AAAATGTAAA
  61 TATTGTATCT CTAATCGTGG AGATGATGAT AGTTTAAGCA TTAATCTATT TATTCCGACT
 121 ATTAACAAGT CTATATATAT TATTATCGGT TTACGGATGA AAAATTTTTG GAAGCCTAAA
 181 TTCGAAATAG AATAATGTTT TTATATTATA CATGTTCTAA AAGAATAATC GATACAGTTT
 241 AAGTGAAAGC TAGAGAGGGG TTTTTAAATG GTCATCGGTC TAGTCATATT CGTGTCTGTG
 301 GCGGCCGCCA TCGTCGGTGT GTTGTCTAAC GTATTGGACA TGCTTATGTA CGTAGAAGAA
 361 AATAATGAAG AGGATGCTAG AATCAAGGAG GAGCAAGAAC TACTGTTGCT ATATTGATAC
 421 ATAATTGAAA ATCTACCAAC TTAAATACAC CGCCTATAAA TTTACAATGA AACACAGATT
 481 GTATTCTGAA GGATTGAGTA TTAGTAATGA TTTAAACTCG ATAATCGGTC AACAATCTAC
 541 AATGGATACG GATATAGAAA TAGACGAAGA TGACATCATG GAACTTCTTA ATATATTGAC
 601 TGAGTTAGGT TGTGATGTCG ACTTTGATGA AAATTTTAGC GATATAGCCG ATGATATTCT
 661 AGAATCGTTG ATAGAACAGG ATGTATAAGT TTTTATGTTA ACTAAATGTG GCCATTTGCA
 721 TCGGTACCTG CGGGAGCAAA ATGTAGGCTG GTAGAAACAC TACCAGAAAA TATGGATTTT
 781 AGATCCGATC ATTTAACAAC ATTTGAATGT TTTAACGAAA TTATCACTCT AGCTAAGAAA
 841 TATATATACA TAGCATCTTT TTGTTGTAAT CCTCTGAGTA CGACTAGGGG AGCGCTTATT
 901 TTTGATAAAC TAAAAGAGGC ATCTGAAAAA GGGATTAAAA TAATAGTTTT GCTAGATGAA
 961 CGAGGGAAAA GAAATCTGGG AGAGCTACAA AGTCACTGCC CGGATATAAA TTTTATAACC
1021 GTTAATATAG ATAAAAAAAA TAATGTGGGA CTACTACTCG GTTGTTTTTG GGTGTCAGAT
1081 GATGAAAGAT GTTATGTAGG AAACGCGTCA TTTACTGGAG GATCTATACA TACGATTAAA
1141 ACGTTAGGTG TATATTCTGA TTATCCCCCG CTGGCCACAG ATCTTCGTAG AAGATTTGAT
1201 ACTTTTAAAG CCTTTAATAG CGCAAAAAAT TCATGGTTGA ATTTATGCTC TGCGGCTTGT
1261 TGTTTGCCAG TTAGCACTGC GTATCATATT AAGAATCCTA TAGGTGGAGT GTTCTTTACT
1321 GATTCTCCGG AACACCTATT GGGATATTCT AGAGATCTAG ATACCGATGT AGTTATTGAT
1381 AAACTCAAGT CGGCTAAGAC TAGTATAGAT ATTGAACATT TGGCCATAGT TCCCACTACA
1441 CGTGTCGACG GTAATAGCTA CTATTGGCCC GACATTTACA ACTCCATTAT AGAAGCAGCC
1501 ATTAATAGAG GAGTTAAGAT CAGACTTCTA GTTGGTAATT GGGATAAGAA CGACGTATAT
1561 TCTATGGCAA CCGCCAGAAG TCTAGACGCG TTGTGTGTTC AAAATGATCT ATCTGTGAAG
1621 GTTTTCACTA TTCAGAATAA TACAAAATTG TTGATAGTCG ACGACGAATA TGTTCATATC
1681 ACTTCGGCAA ATTTCGACGG AACCCATTAC CAAAATCACG GATTCGTCAG TTTTAATAGT
1741 ATAGATAAAC AGCTTGTAAG CGAGGCTAAA AAAATATTTG AGAGAGATTG GGTGAGCTCA
1801 CATTCAAAGA GTCTTAAGAT ATAACTCGAG AAAAATTGAA ATTTTATTTT TTTTTTTTGG
1861 AATATAAATA AGGAATTCCT GCAGGCTAGC CCCGGGAAGC TTCCATGGAG GCCTAAATAA
1921 ATAATTTTTA TGGATCCGGA GAGCTCGGGT ATCTAGCCAC AGTAAATCGT TAAAAATTTA
1981 AAAAAAAGAA AATAGAGACG TATAGAACGC CATCATGTTA AACAGGGTAC AAATCTTGAT
2041 GAAAACAGCT AACAATTATG AAACTATTGA GATATTGCGT AATTATTTAA GACTGTATAT
2101 TATTTTGGCA CGAAATGAAG AAGGCCGTGG TATACTAATA TACGATGATA ACATAGATAG
```

```
2161 TATTATGTCG ATGATGAATA TTACAAGATT AGAAGTTATA GGATTGACGA CTCATTGCAC
2221 AAAATTAAGA TCATCGCCTC CAATTCCTAT GTCTAGATTG TTTATGGACG AAATAGATCA
2281 TGAGTCATAT TATTCTCCAA AAACTTCAGA TTATCCGTTG ATCGATATTA TACGAAAGCG
2341 TTCCCACGAA CAGGGAGATA TAGCACTGGC TTTAGAACAA TACGGTATCG AGAATACAGA
2401 TTCCATATCA GAAATTAATG AATGGCTGTC GTCAAAAGGT TTAGCATGTT ATAGATTTGT
2461 AAAATTTAAC GATTATAGGA AACAGATGTA TCGTAAGTTC TCTAGGTGTA CTATAGTTGA
2521 CAGTATGATA ATAGGGCATA TAGGTCATCA TTATATTTGG ATTAAAAATT TAGAAACATA
2581 TACGCGTCCC GAAATTGATG TGTTACCGTT TGATATTAAA TACATATCTA GAGATGCATA
2641 GCTTGAGTAT TCTATAGTGT CACCTAAATA GCTTGGCGTA ATCATGGTCA TAGCTGTTTC
2701 CTGTGTGAAA TTGTTATCCG CTCACAATTC CACACAACAT ACGAGCCGGA AGCATAAAGT
2761 GTAAAGCCTG GGGTGCCTAA TGAGTGAGCT AACTCACATT AATTGCGTTG CGCTCACTGC
2821 CCGCTTTCCA GTCGGGAAAC CTGTCGTGCC AGCTGCATTA ATGAATCGGC CAACGCGCGG
2881 GGAGAGGCGG TTTGCGTATT GGGCGCTCTT CCGCTTCCTC GCTCACTGAC TCGCTGCGCT
2941 CGGTCGTTCG GCTGCGGCGA GCGGTATCAG CTCACTCAAA GGCGGTAATA CGGTTATCCA
3001 CAGAATCAGG GGATAACGCA GGAAAGAACA TGTGAGCAAA AGGCCAGCAA AAGGCCAGGA
3061 ACCGTAAAAA GGCCGCGTTG CTGGCGTTTT TCGATAGGCT CCGCCCCCCT GACGAGCATC
3121 ACAAAAATCG ACGCTCAAGT CAGAGGTGGC GAAACCCGAC AGGACTATAA AGATACCAGG
3181 CGTTTCCCCC TGGAAGCTCC CTCGTGCGCT CTCCTGTTCC GACCCTGCCG CTTACCGGAT
3241 ACCTGTCCGC CTTTCTCCCT TCGGGAAGCG TGGCGCTTTC TCATAGCTCA CGCTGTAGGT
3301 ATCTCAGTTC GGTGTAGGTC GTTCGCTCCA AGCTGGGCTG TGTGCACGAA CCCCCCGTTC
3361 AGCCCGACCG CTGCGCCTTA TCCGGTAACT ATCGTCTTGA GTCCAACCCG GTAAGACACG
3421 ACTTATCGCC ACTGGCAGCA GCCACTGGTA ACAGGATTAG CAGAGCGAGG TATGTAGGCG
3481 GTGCTACAGA GTTCTTGAAG TGGTGGCCTA ACTACGGCTA CACTAGAAGG ACAGTATTTG
3541 GTATCTGCGC TCTGCTGAAG CCAGTTACCT TCGGAAAAAG AGTTGGTAGC TCTTGATCCG
3601 GCAAACAAAC CACCGCTGGT AGCGGTGGTT TTTTTGTTTG CAAGCAGCAG ATTACGCGCA
3661 GAAAAAAAGG ATCTCAAGAA GATCCTTTGA TCTTTTCTAC GGGGTCTGAC GCTCAGTGGA
3721 ACGAAAACTC ACGTTAAGGG ATTTTGGTCA TGAGATTATC AAAAAGGATC TTCACCTAGA
3781 TCCTTTTAAA TTAAAAATGA AGTTTTAAAT CAATCTAAAG TATATATGAG TAAACTTGGT
3841 CTGACAGTTA CCAATGCTTA ATCAGTGAGG CACCTATCTC AGCGATCTGT CTATTTCGTT
3901 CATCCATAGT TGCCTGACTC CCCGTCGTGT AGATAACTAC GATACGGGAG GGCTTACCAT
3961 CTGGCCCCAG TGCTGCAATG ATACCGCGAG ACCCACGCTC ACCGGCTCCA GATTTATCAG
4021 CAATAAACCA GCCAGCCGGA AGGGCCGAGC GCAGAAGTGG TCCTGCAACT TTATCCGCCT
4081 CCATCCAGTC TATTAATTGT TGCCGGGAAG CTAGAGTAAG TAGTTCGCCA GTTAATAGTT
4141 TGCGCAACGT TGTTGGCATT GCTACAGGCA TCGTGGTGTC ACGCTCGTCG TTTGGTATGG
4201 CTTCATTCAG CTCCGGTTCC CAACGATCAA GGCGAGTTAC ATGATCCCCC ATGTTGTGCA
4261 AAAAAGCGGT TAGCTCCTTC GGTCCTCCGA TCGTTGTCAG AAGTAAGTTG GCCGCAGTGT
4321 TATCACTCAT GGTTATGGCA GCACTGCATA ATTCTCTTAC TGTCATGCCA TCCGTAAGAT
4381 GCTTTTCTGT GACTGGTGAG TACTCAACCA AGTCATTCTG AGAATACCGC GCCCGGCGAC
4441 CGAGTTGCTC TTGCCCGGCG TCAATACGGG ATAATAGTGT ATGACATAGC AGAACTTTAA
4501 AAGTGCTCAT CATTGGAAAA CGTTCTTCGG GGCGAAAACT CTCAAGGATC TTACCGCTGT
4561 TGAGATCCAG TTCGATGTAA CCCACTCGTG CACCCAACTG ATCTTCAGCA TCTTTTACTT
4621 TCACCAGCGT TTCTGGGTGA GCAAAAACAG GAAGGCAAAA TGCCGCAAAA AAGGGAATAA
4681 GGGCGACACG GAAATGTTGA ATACTCATAC TCTTCCTTTT TCAATATTAT TGAAGCATTT
4741 ATCAGGGTTA TTGTCTCATG AGCGGATACA TATTTGAATG TATTTAGAAA AATAAACAAA
4801 TAGGGGTTCC GCGCACATTT CCCCGAAAAG TGCCACCTGT ATGCGGTGTG AAATACCGCA
4861 CAGATGCGTA AGGAGAAAAT ACCGCATCAG GCGAAATTGT AAACGTTAAT ATTTTGTTAA
4921 AATTCGCGTT AAATATTTGT TAAATCAGCT CATTTTTTAA CCAATAGGCC GAAATCGGCA
4981 AAATCCCTTA TAAATCAAAA GAATAGACCG AGATAGGGTT GAGTGTTGTT CCAGTTTGGA
5041 ACAAGAGTCC ACTATTAAAG AACGTGGACT CCAACGTCAA AGGGCGAAAA ACCGTCTATC
5101 AGGGCGATGG CCCACTACGT GAACCATCAC CCAAATCAAG TTTTTTGCGG TCGAGGTGCC
5161 GTAAAGCTCT AAATCGGAAC CCTAAAGGGA GCCCCCGATT TAGAGCTTGA CGGGGAAAGC
5221 CGGCGAACGT GGCGAGAAAG GAAGGGAAGA AAGCGAAAGG AGCGGGCGCT AGGGCGCTGG
5281 CAAGTGTAGC GGTCACGCTG CGCGTAACCA CCACACCCGC CGCGCTTAAT GCGCCGCTAC
5341 AGGGCGCGTC CATTCGCCAT TCAGGCTGCG CAACTGTTGG GAAGGGCGAT CGGTGCGGGC
5401 CTCTTCGCTA TTACGCCAGC TGGCGAAAGG GGGATGTGCT GCAAGGCGAT TAAGTTGGGT
5461 AACGCCAGGG TTTTCCCAGT CACGACGTTG TAAAACGACG GCCAGTGAAT TGTAATACGA
5521 CTCACTATA
//
```

pRB-Scarlet (pRB-mScarlet-i-NES)

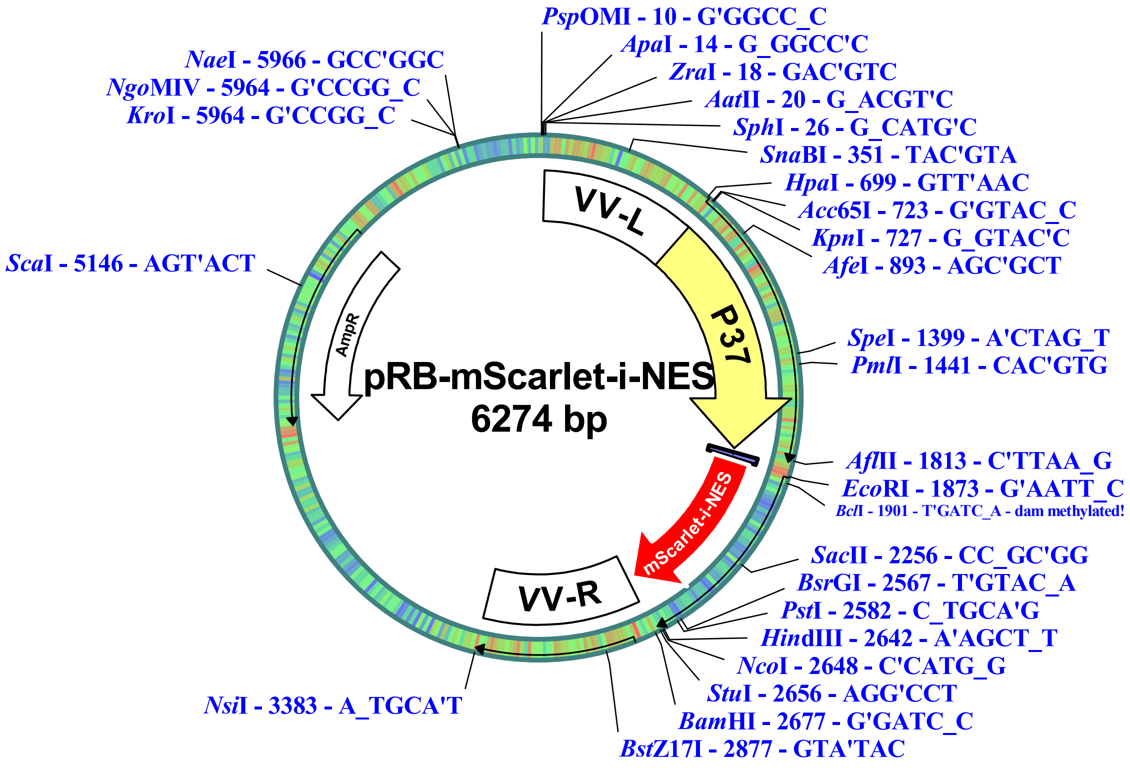


```
   1 GGGCGAATTG GGCCCGACGT CGCATGCGAT AAAGTTTCGA AACAGCAAAA AAAATGTAAA
  61 TATTGTATCT CTAATCGTGG AGATGATGAT AGTTTAAGCA TTAATCTATT TATTCCGACT
 121 ATTAACAAGT CTATATATAT TATTATCGGT TTACGGATGA AAAATTTTTG GAAGCCTAAA
 181 TTCGAAATAG AATAATGTTT TTATATTATA CATGTTCTAA AAGAATAATC GATACAGTTT
 241 AAGTGAAAGC TAGAGAGGGG TTTTTAAATG GTCATCGGTC TAGTCATATT CGTGTCTGTG
 301 GCGGCCGCCA TCGTCGGTGT GTTGTCTAAC GTATTGGACA TGCTTATGTA CGTAGAAGAA
 361 AATAATGAAG AGGATGCTAG AATCAAGGAG GAGCAAGAAC TACTGTTGCT ATATTGATAC
 421 ATAATTGAAA ATCTACCAAC TTAAATACAC CGCCTATAAA TTTACAATGA AACACAGATT
 481 GTATTCTGAA GGATTGAGTA TTAGTAATGA TTTAAACTCG ATAATCGGTC AACAATCTAC
 541 AATGGATACG GATATAGAAA TAGACGAAGA TGACATCATG GAACTTCTTA ATATATTGAC
 601 TGAGTTAGGT TGTGATGTCG ACTTTGATGA AAATTTTAGC GATATAGCCG ATGATATTCT
 661 AGAATCGTTG ATAGAACAGG ATGTATAAGT TTTTATGTTA ACTAAATGTG GCCATTTGCA
 721 TCGGTACCTG CGGGAGCAAA ATGTAGGCTG GTAGAAACAC TACCAGAAAA TATGGATTTT
 781 AGATCCGATC ATTTAACAAC ATTTGAATGT TTTAACGAAA TTATCACTCT AGCTAAGAAA
 841 TATATATACA TAGCATCTTT TTGTTGTAAT CCTCTGAGTA CGACTAGGGG AGCGCTTATT
 901 TTTGATAAAC TAAAAGAGGC ATCTGAAAAA GGGATTAAAA TAATAGTTTT GCTAGATGAA
 961 CGAGGGAAAA GAAATCTGGG AGAGCTACAA AGTCACTGCC CGGATATAAA TTTTATAACC
1021 GTTAATATAG ATAAAAAAAA TAATGTGGGA CTACTACTCG GTTGTTTTTG GGTGTCAGAT
1081 GATGAAAGAT GTTATGTAGG AAACGCGTCA TTTACTGGAG GATCTATACA TACGATTAAA
1141 ACGTTAGGTG TATATTCTGA TTATCCCCCG CTGGCCACAG ATCTTCGTAG AAGATTTGAT
1201 ACTTTTAAAG CCTTTAATAG CGCAAAAAAT TCATGGTTGA ATTTATGCTC TGCGGCTTGT
1261 TGTTTGCCAG TTAGCACTGC GTATCATATT AAGAATCCTA TAGGTGGAGT GTTCTTTACT
1321 GATTCTCCGG AACACCTATT GGGATATTCT AGAGATCTAG ATACCGATGT AGTTATTGAT
1381 AAACTCAAGT CGGCTAAGAC TAGTATAGAT ATTGAACATT TGGCCATAGT TCCCACTACA
1441 CGTGTCGACG GTAATAGCTA CTATTGGCCC GACATTTACA ACTCCATTAT AGAAGCAGCC
1501 ATTAATAGAG GAGTTAAGAT CAGACTTCTA GTTGGTAATT GGGATAAGAA CGACGTATAT
1561 TCTATGGCAA CCGCCAGAAG TCTAGACGCG TTGTGTGTTC AAAATGATCT ATCTGTGAAG
1621 GTTTTCACTA TTCAGAATAA TACAAAATTG TTGATAGTCG ACGACGAATA TGTTCATATC
1681 ACTTCGGCAA ATTTCGACGG AACCCATTAC CAAAATCACG GATTCGTCAG TTTTAATAGT
1741 ATAGATAAAC AGCTTGTAAG CGAGGCTAAA AAAATATTTG AGAGAGATTG GGTGAGCTCA
1801 CATTCAAAGA GTCTTAAGAT ATAACTCGAG AAAAATTGAA ATTTTATTTT TTTTTTTTGG
1861 AATATAAATA AGGAATTCAT GGTGAGCAAG GGCGAGGCAG TGATCAAGGA GTTCATGCGG
1921 TTCAAGGTGC ACATGGAGGG CTCCATGAAC GGCCACGAGT TCGAGATCGA GGGCGAGGGC
1981 GAGGGCCGCC CCTACGAGGG CACCCAGACC GCCAAGCTGA AGGTGACCAA GGGTGGCCCC
2041 CTGCCCTTCT CCTGGGACAT CCTGTCCCCT CAGTTCATGT ACGGCTCCAG GGCCTTCATC
```

2101 AAGCACCCCG CCGACATCCC CGACTACTAT AAGCAGTCCT TCCCCGAGGG CTTCAAGTGG
2161 GAGCGCGTGA TGAACTTCGA GGACGGCGGC GCCGTGACCG TGACCCAGGA CACCTCCCTG
2221 GAGGACGGCA CCCTGATCTA CAAGGTGAAG CTCCGCGGCA CCAACTTCCC TCCTGACGGC
2281 CCCGTAATGC AGAAGAAGAC AATGGGCTGG GAAGCATCCA CCGAGCGGTT GTACCCCGAG
2341 GACGGCGTGC TGAAGGGCGA CATTAAGATG GCCCTGCGCC TGAAGGACGG CGGCCGCTAC
2401 CTGGCGGACT TCAAGACCAC CTACAAGGCC AAGAAGCCCG TGCAGATGCC CGGCGCCTAC
2461 AACGTCGACC GCAAGTTGGA CATCACCTCC CACAACGAGG ACTACACCGT GGTGGAACAG
2521 TACGAACGCT CCGAGGGCCG CCACTCCACC GGCGGCATGG ACGAGCTGTA CAGTGAGCTG
2581 CAGAACAAGC TGGAAGAGTT GGATCTGGAC TCGTACAAGT CCGGACTCAG ATCTCGAGCT
2641 CAAGCTTCCA TGGAGGCCTA AATAAATAAT TTTTATGGAT CCGGAGAGCT CGGGTATCTA
2701 GCCACAGTAA ATCGTTAAAA ATTTAAAAAA AAGAAAATAG AGACGTATAG AACGCCATCA
2761 TGTTAAACAG GGTACAAATC TTGATGAAAA CAGCTAACAA TTATGAAACT ATTGAGATAT
2821 TGCGTAATTA TTTAAGACTG TATATTATTT TGGCACGAAA TGAAGAAGGC CGTGGTATAC
2881 TAATATACGA TGATAACATA GATAGTATTA TGTCGATGAT GAATATTACA AGATTAGAAG
2941 TTATAGGATT GACGACTCAT TGCACAAAAT TAAGATCATC GCCTCCAATT CCTATGTCTA
3001 GATTGTTTAT GGACGAAATA GATCATGAGT CATATTATTC TCCAAAAACT TCAGATTATC
3061 CGTTGATCGA TATTATACGA AAGCGTTCCC ACGAACAGGG AGATATAGCA CTGGCTTTAG
3121 AACAATACGG TATCGAGAAT ACAGATTCCA TATCAGAAAT TAATGAATGG CTGTCGTCAA
3181 AAGGTTTAGC ATGTTATAGA TTTGTAAAAT TTAACGATTA TAGGAAACAG ATGTATCGTA
3241 AGTTCTCTAG GTGTACTATA GTTGACAGTA TGATAATAGG GCATATAGGT CATCATTATA
3301 TTTGGATTAA AAATTTAGAA ACATATACGC GTCCCGAAAT TGATGTGTTA CCGTTTGATA
3361 TTAAATACAT ATCTAGAGAT GCATAGCTTG AGTATTCTAT AGTGTCACCT AAATAGCTTG
3421 GCGTAATCAT GGTCATAGCT GTTTCCTGTG TGAAATTGTT ATCCGCTCAC AATTCCACAC
3481 AACATACGAG CCGGAAGCAT AAAGTGTAAA GCCTGGGGTG CCTAATGAGT GAGCTAACTC
3541 ACATTAATTG CGTTGCGCTC ACTGCCCGCT TTCCAGTCGG GAAACCTGTC GTGCCAGCTG
3601 CATTAATGAA TCGGCCAACG CGCGGGGAGA GGCGGTTTGC GTATTGGGCG CTCTTCCGCT
3661 TCCTCGCTCA CTGACTCGCT GCGCTCGGTC GTTCGGCTGC GGCGAGCGGT ATCAGCTCAC
3721 TCAAAGGCGG TAATACGGTT ATCCACAGAA TCAGGGGATA ACGCAGGAAA GAACATGTGA
3781 GCAAAAGGCC AGCAAAAGGC CAGGAACCGT AAAAAGGCCG CGTTGCTGGC GTTTTTCGAT
3841 AGGCTCCGCC CCCCTGACGA GCATCACAAA AATCGACGCT CAAGTCAGAG GTGGCGAAAC
3901 CCGACAGGAC TATAAAGATA CCAGGCGTTT CCCCCTGGAA GCTCCCTCGT GCGCTCTCCT
3961 GTTCCGACCC TGCCGCTTAC CGGATACCTG TCCGCCTTTC TCCCTTCGGG AAGCGTGGCG
4021 CTTTCTCATA GCTCACGCTG TAGGTATCTC AGTTCGGTGT AGGTCGTTCG CTCCAAGCTG
4081 GGCTGTGTGC ACGAACCCCC CGTTCAGCCC GACCGCTGCG CCTTATCCGG TAACTATCGT
4141 CTTGAGTCCA ACCCGGTAAG ACACGACTTA TCGCCACTGG CAGCAGCCAC TGGTAACAGG
4201 ATTAGCAGAG CGAGGTATGT AGGCGGTGCT ACAGAGTTCT TGAAGTGGTG GCCTAACTAC
4261 GGCTACACTA GAAGGACAGT ATTTGGTATC TGCGCTCTGC TGAAGCCAGT TACCTTCGGA
4321 AAAAGAGTTG GTAGCTCTTG ATCCGGCAAA CAAACCACCG CTGGTAGCGG TGGTTTTTTT
4381 GTTTGCAAGC AGCAGATTAC GCGCAGAAAA AAAGGATCTC AAGAAGATCC TTTGATCTTT
4441 TCTACGGGGT CTGACGCTCA GTGGAACGAA AACTCACGTT AAGGGATTTT GGTCATGAGA
4501 TTATCAAAAA GGATCTTCAC CTAGATCCTT TTAAATTAAA AATGAAGTTT TAAATCAATC
4561 TAAAGTATAT ATGAGTAAAC TTGGTCTGAC AGTTACCAAT GCTTAATCAG TGAGGCACCT
4621 ATCTCAGCGA TCTGTCTATT TCGTTCATCC ATAGTTGCCT GACTCCCCGT CGTGTAGATA
4681 ACTACGATAC GGGAGGGCTT ACCATCTGGC CCCAGTGCTG CAATGATACC GCGAGACCCA
4741 CGCTCACCGG CTCCAGATTT ATCAGCAATA AACCAGCCAG CCGGAAGGGC CGAGCGCAGA
4801 AGTGGTCCTG CAACTTTATC CGCCTCCATC CAGTCTATTA ATTGTTGCCG GGAAGCTAGA
4861 GTAAGTAGTT CGCCAGTTAA TAGTTTGCGC AACGTTGTTG GCATTGCTAC AGGCATCGTG
4921 GTGTCACGCT CGTCGTTTGG TATGGCTTCA TTCAGCTCCG GTTCCCAACG ATCAAGGCGA
4981 GTTACATGAT CCCCCATGTT GTGCAAAAAA GCGGTTAGCT CCTTCGGTCC TCCGATCGTT
5041 GTCAGAAGTA AGTTGGCCGC AGTGTTATCA CTCATGGTTA TGGCAGCACT GCATAATTCT
5101 CTTACTGTCA TGCCATCCGT AAGATGCTTT TCTGTGACTG GTGAGTACTC AACCAAGTCA
5161 TTCTGAGAAT ACCGCGCCCG GCGACCGAGT TGCTCTTGCC CGGCGTCAAT ACGGGATAAT
5221 AGTGTATGAC ATAGCAGAAC TTTAAAAGTG CTCATCATTG GAAAACGTTC TTCGGGGCGA
5281 AAACTCTCAA GGATCTTACC GCTGTTGAGA TCCAGTTCGA TGTAACCCAC TCGTGCACCC
5341 AACTGATCTT CAGCATCTTT TACTTTCACC AGCGTTTCTG GGTGAGCAAA AACAGGAAGG
5401 CAAAATGCCG CAAAAAAGGG AATAAGGGCG ACACGGAAAT GTTGAATACT CATACTCTTC
5461 CTTTTTCAAT ATTATTGAAG CATTTATCAG GGTTATTGTC TCATGAGCGG ATACATATTT
5521 GAATGTATTT AGAAAAATAA ACAAATAGGG GTTCCGCGCA CATTTCCCCG AAAAGTGCCA
5581 CCTGTATGCG GTGTGAAATA CCGCACAGAT GCGTAAGGAG AAAATACCGC ATCAGGCGAA
5641 ATTGTAAACG TTAATATTTT GTTAAAATTC GCGTTAAATA TTTGTTAAAT CAGCTCATTT
5701 TTTAACCAAT AGGCCGAAAT CGGCAAAATC CCTTATAAAT CAAAAGAATA GACCGAGATA
5761 GGGTTGAGTG TTGTTCCAGT TTGGAACAAG AGTCCACTAT TAAAGAACGT GGACTCCAAC
5821 GTCAAAGGGC GAAAAACCGT CTATCAGGGC GATGGCCCAC TACGTGAACC ATCACCCAAA
5881 TCAAGTTTTT TGCGGTCGAG GTGCCGTAAA GCTCTAAATC GGAACCCTAA AGGGAGCCCC
5941 CGATTTAGAG CTTGACGGGG AAAGCCGGCG AACGTGGCGA GAAAGGAAGG GAAGAAAGCG
6001 AAAGGAGCGG GCGCTAGGGC GCTGGCAAGT GTAGCGGTCA CGCTGCGCGT AACCACCACA
6061 CCCGCCGCGC TTAATGCGCC GCTACAGGGC GCGTCCATTC GCCATTCAGG CTGCGCAACT
6121 GTTGGGAAGG GCGATCGGTG CGGGCCTCTT CGCTATTACG CCAGCTGGCG AAAGGGGGAT
6181 GTGCTGCAAG GCGATTAAGT TGGGTAACGC CAGGGTTTTC CCAGTCACGA CGTTGTAAAA
6241 CGACGGCCAG TGAATTGTAA TACGACTCAC TATA

pRB-TagGFP2

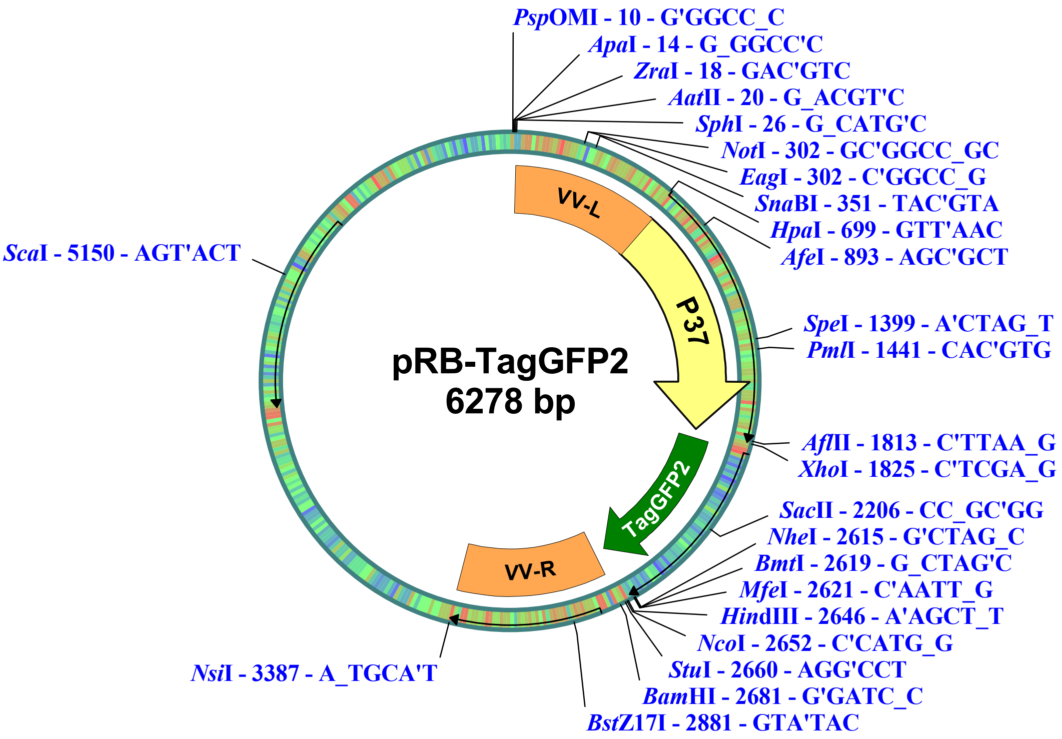


```
   1 GGGCGAATTG GGCCCGACGT CGCATGCGAT AAAGTTTCGA AACAGCAAAA AAAATGTAAA
  61 TATTGTATCT CTAATCGTGG AGATGATGAT AGTTTAAGCA TTAATCTATT TATTCCGACT
 121 ATTAACAAGT CTATATATAT TATTATCGGT TTACGGATGA AAAATTTTTG GAAGCCTAAA
 181 TTCGAAATAG AATAATGTTT TTATATTATA CATGTTCTAA AAGAATAATC GATACAGTTT
 241 AAGTGAAAGC TAGAGAGGGG TTTTTAAATG GTCATCGGTC TAGTCATATT CGTGTCTGTG
 301 GCGGCCGCCA TCGTCGGTGT GTTGTCTAAC GTATTGGACA TGCTTATGTA CGTAGAAGAA
 361 AATAATGAAG AGGATGCTAG AATCAAGGAG GAGCAAGAAC TACTGTTGCT ATATTGATAC
 421 ATAATTGAAA ATCTACCAAC TTAAATACAC CGCCTATAAA TTTACAATGA AACACAGATT
 481 GTATTCTGAA GGATTGAGTA TTAGTAATGA TTTAAACTCG ATAATCGGTC AACAATCTAC
 541 AATGGATACG GATATAGAAA TAGACGAAGA TGACATCATG GAACTTCTTA ATATATTGAC
 601 TGAGTTAGGT TGTGATGTCG ACTTTGATGA AAATTTTAGC GATATAGCCG ATGATATTCT
 661 AGAATCGTTG ATAGAACAGG ATGTATAAGT TTTTATGTTA ACTAAATGTG GCCATTTGCA
 721 TCGGTACCTG CGGGAGCAAA ATGTAGGCTG GTAGAAACAC TACCAGAAAA TATGGATTTT
 781 AGATCCGATC ATTTAACAAC ATTTGAATGT TTTAACGAAA TTATCACTCT AGCTAAGAAA
 841 TATATATACA TAGCATCTTT TTGTTGTAAT CCTCTGAGTA CGACTAGGGG AGCGCTTATT
 901 TTTGATAAAC TAAAAGAGGC ATCTGAAAAA GGGATTAAAA TAATAGTTTT GCTAGATGAA
 961 CGAGGGAAAA GAAATCTGGG AGAGCTACAA AGTCACTGCC CGGATATAAA TTTTATAACC
1021 GTTAATATAG ATAAAAAAAA TAATGTGGGA CTACTACTCG GTTGTTTTTG GGTGTCAGAT
1081 GATGAAAGAT GTTATGTAGG AAACGCGTCA TTTACTGGAG GATCTATACA TACGATTAAA
1141 ACGTTAGGTG TATATTCTGA TTATCCCCCG CTGGCCACAG ATCTTCGTAG AAGATTTGAT
1201 ACTTTTAAAG CCTTTAATAG CGCAAAAAAT TCATGGTTGA ATTTATGCTC TGCGGCTTGT
1261 TGTTTGCCAG TTAGCACTGC GTATCATATT AAGAATCCTA TAGGTGGAGT GTTCTTTACT
1321 GATTCTCCGG AACACCTATT GGGATATTCT AGAGATCTAG ATACCGATGT AGTTATTGAT
1381 AAACTCAAGT CGGCTAAGAC TAGTATAGAT ATTGAACATT TGGCCATAGT TCCCACTACA
1441 CGTGTCGACG GTAATAGCTA CTATTGGCCC GACATTTACA ACTCCATTAT AGAAGCAGCC
1501 ATTAATAGAG GAGTTAAGAT CAGACTTCTA GTTGGTAATT GGGATAAGAA CGACGTATAT
1561 TCTATGGCAA CCGCCAGAAG TCTAGACGCG TTGTGTGTTC AAAATGATCT ATCTGTGAAG
1621 GTTTTCACTA TTCAGAATAA TACAAAATTG TTGATAGTCG ACGACGAATA TGTTCATATC
1681 ACTTCGGCAA ATTTCGACGG AACCCATTAC CAAAATCACG GATTCGTCAG TTTTAATAGT
1741 ATAGATAAAC AGCTTGTAAG CGAGGCTAAA AAAATATTTG AGAGAGATTG GGTGAGCTCA
1801 CATTCAAAGA GTCTTAAGAT ATAACTCGAG AAAAATTGAA ATTTTATTTT TTTTTTTTGG
1861 AATATAAATG AGCGGTACCG GAAGCGGGGG CGAGGAGCTG TTCGCCGGCA TCGTGCCCGT
1921 GCTGATCGAG CTGGACGGCG ACGTGCACGG CCACAAGTTC AGCGTGCGCG GCGAGGGCGA
1981 GGGCGACGCC GACTACGGCA AGCTGGAGAT CAAGTTCATC TGCACCACCG GCAAGCTGCC
2041 CGTGCCCTGG CCCACCCTGG TGACCACCCT CTGCTACGGC ATCCAGTGCT TCGCCCGCTA
```

2101 CCCCGAGCAC ATGAAGATGA ACGACTTCTT CAAGAGCGCC ATGCCCGAGG GCTACATCCA
2161 GGAGCGCACC ATCCAGTTCC AGGACGACGG CAAGTACAAG ACCCGCGGCG AGGTGAAGTT
2221 CGAGGGCGAC ACCCTGGTGA ACCGCATCGA GCTGAAGGGC AAGGACTTCA AGGAGGACGG
2281 CAACATCCTG GGCCACAAGC TGGAGTACAG CTTCAACAGC CACAACGTGT ACATCCGCCC
2341 CGACAAGGCC AACAACGGCC TGGAGGCTAA CTTCAAGACC CGCCACAACA TCGAGGGCGG
2401 CGGCGTGCAG CTGGCCGACC ACTACCAGAC CAACGTGCCC CTGGGCGACG GCCCCGTGCT
2461 GATCCCCATC AACCACTACC TGAGCACTCA GACCAAGATC AGCAAGGACC GCAACGAGGC
2521 CCGCGACCAC ATGGTGCTCC TGGAGTCCTT CAGCGCCTGC TGCCACACCC ACGGCATGGA
2581 CGAGCTGTAC AGGTCCGGAG GTGGAGGTTC CGGAGCTAGC CAATTGTTAT AATTTTTCTT
2641 TTTCTAAGCT TCCATGGAGG CCTAAATAAA TAATTTTTAT GGATCCGGAG AGCTCGGGTA
2701 TCTAGCCACA GTAAATCGTT AAAAATTTAA AAAAAAGAAA ATAGAGACGT ATAGAACGCC
2761 ATCATGTTAA ACAGGGTACA AATCTTGATG AAAACAGCTA ACAATTATGA AACTATTGAG
2821 ATATTGCGTA ATTATTTAAG ACTGTATATT ATTTTGGCAC GAAATGAAGA AGGCCGTGGT
2881 ATACTAATAT ACGATGATAA CATAGATAGT ATTATGTCGA TGATGAATAT TACAAGATTA
2941 GAAGTTATAG GATTGACGAC TCATTGCACA AAATTAAGAT CATCGCCTCC AATTCCTATG
3001 TCTAGATTGT TTATGGACGA AATAGATCAT GAGTCATATT ATTCTCCAAA AACTTCAGAT
3061 TATCCGTTGA TCGATATTAT ACGAAAGCGT TCCCACGAAC AGGGAGATAT AGCACTGGCT
3121 TTAGAACAAT ACGGTATCGA GAATACAGAT TCCATATCAG AAATTAATGA ATGGCTGTCG
3181 TCAAAAGGTT TAGCATGTTA TAGATTTGTA AAATTTAACG ATTATAGGAA ACAGATGTAT
3241 CGTAAGTTCT CTAGGTGTAC TATAGTTGAC AGTATGATAA TAGGGCATAT AGGTCATCAT
3301 TATATTTGGA TTAAAAATTT AGAAACATAT ACGCGTCCCG AAATTGATGT GTTACCGTTT
3361 GATATTAAAT ACATATCTAG AGATGCATAG CTTGAGTATT CTATAGTGTC ACCTAAATAG
3421 CTTGGCGTAA TCATGGTCAT AGCTGTTTCC TGTGTGAAAT TGTTATCCGC TCACAATTCC
3481 ACACAACATA CGAGCCGGAA GCATAAAGTG TAAAGCCTGG GGTGCCTAAT GAGTGAGCTA
3541 ACTCACATTA ATTGCGTTGC GCTCACTGCC CGCTTTCCAG TCGGGAAACC TGTCGTGCCA
3601 GCTGCATTAA TGAATCGGCC AACGCGCGGG GAGAGGCGGT TTGCGTATTG GGCGCTCTTC
3661 CGCTTCCTCG CTCACTGACT CGCTGCGCTC GGTCGTTCGG CTGCGGCGAG CGGTATCAGC
3721 TCACTCAAAG GCGGTAATAC GGTTATCCAC AGAATCAGGG GATAACGCAG GAAAGAACAT
3781 GTGAGCAAAA GGCCAGCAAA AGGCCAGGAA CCGTAAAAAG GCCGCGTTGC TGGCGTTTTT
3841 CGATAGGCTC CGCCCCCCTG ACGAGCATCA CAAAAATCGA CGCTCAAGTC AGAGGTGGCG
3901 AAACCCGACA GGACTATAAA GATACCAGGC GTTTCCCCCT GGAAGCTCCC TCGTGCGCTC
3961 TCCTGTTCCG ACCCTGCCGC TTACCGGATA CCTGTCCGCC TTTCTCCCTT CGGGAAGCGT
4021 GGCGCTTTCT CATAGCTCAC GCTGTAGGTA TCTCAGTTCG GTGTAGGTCG TTCGCTCCAA
4081 GCTGGGCTGT GTGCACGAAC CCCCCGTTCA GCCCGACCGC TGCGCCTTAT CCGGTAACTA
4141 TCGTCTTGAG TCCAACCCGG TAAGACACGA CTTATCGCCA CTGGCAGCAG CCACTGGTAA
4201 CAGGATTAGC AGAGCGAGGT ATGTAGGCGG TGCTACAGAG TTCTTGAAGT GGTGGCCTAA
4261 CTACGGCTAC ACTAGAAGGA CAGTATTTGG TATCTGCGCT CTGCTGAAGC CAGTTACCTT
4321 CGGAAAAAGA GTTGGTAGCT CTTGATCCGG CAAACAAACC ACCGCTGGTA GCGGTGGTTT
4381 TTTTGTTTGC AAGCAGCAGA TTACGCGCAG AAAAAAAGGA TCTCAAGAAG ATCCTTTGAT
4441 CTTTTCTACG GGGTCTGACG CTCAGTGGAA CGAAAACTCA CGTTAAGGGA TTTTGGTCAT
4501 GAGATTATCA AAAAGGATCT TCACCTAGAT CCTTTTAAAT TAAAAATGAA GTTTTAAATC
4561 AATCTAAAGT ATATATGAGT AAACTTGGTC TGACAGTTAC CAATGCTTAA TCAGTGAGGC
4621 ACCTATCTCA GCGATCTGTC TATTTCGTTC ATCCATAGTT GCCTGACTCC CCGTCGTGTA
4681 GATAACTACG ATACGGGAGG GCTTACCATC TGGCCCCAGT GCTGCAATGA TACCGCGAGA
4741 CCCACGCTCA CCGGCTCCAG ATTTATCAGC AATAAACCAG CCAGCCGGAA GGGCCGAGCG
4801 CAGAAGTGGT CCTGCAACTT TATCCGCCTC CATCCAGTCT ATTAATTGTT GCCGGGAAGC
4861 TAGAGTAAGT AGTTCGCCAG TTAATAGTTT GCGCAACGTT GTTGGCATTG CTACAGGCAT
4921 CGTGGTGTCA CGCTCGTCGT TTGGTATGGC TTCATTCAGC TCCGGTTCCC AACGATCAAG
4981 GCGAGTTACA TGATCCCCCA TGTTGTGCAA AAAAGCGGTT AGCTCCTTCG GTCCTCCGAT
5041 CGTTGTCAGA AGTAAGTTGG CCGCAGTGTT ATCACTCATG GTTATGGCAG CACTGCATAA
5101 TTCTCTTACT GTCATGCCAT CCGTAAGATG CTTTTCTGTG ACTGGTGAGT ACTCAACCAA
5161 GTCATTCTGA GAATACCGCG CCCGGCGACC GAGTTGCTCT TGCCCGGCGT CAATACGGGA
5221 TAATAGTGTA TGACATAGCA GAACTTTAAA AGTGCTCATC ATTGGAAAAC GTTCTTCGGG
5281 GCGAAAACTC TCAAGGATCT TACCGCTGTT GAGATCCAGT TCGATGTAAC CCACTCGTGC
5341 ACCCAACTGA TCTTCAGCAT CTTTTACTTT CACCAGCGTT TCTGGGTGAG CAAAAACAGG
5401 AAGGCAAAAT GCCGCAAAAA AGGGAATAAG GGCGACACGG AAATGTTGAA TACTCATACT
5461 CTTCCTTTTT CAATATTATT GAAGCATTTA TCAGGGTTAT TGTCTCATGA GCGGATACAT
5521 ATTTGAATGT ATTTAGAAAA ATAAACAAAT AGGGGTTCCG CGCACATTTC CCCGAAAAGT
5581 GCCACCTGTA TGCGGTGTGA AATACCGCAC AGATGCGTAA GGAGAAAATA CCGCATCAGG
5641 CGAAATTGTA AACGTTAATA TTTTGTTAAA ATTCGCGTTA AATATTTGTT AAATCAGCTC
5701 ATTTTTTAAC CAATAGGCCG AAATCGGCAA AATCCCTTAT AAATCAAAAG AATAGACCGA
5761 GATAGGGTTG AGTGTTGTTC CAGTTTGGAA CAAGAGTCCA CTATTAAAGA ACGTGGACTC
5821 CAACGTCAAA GGGCGAAAAA CCGTCTATCA GGGCGATGGC CCACTACGTG AACCATCACC
5881 CAAATCAAGT TTTTTGCGGT CGAGGTGCCG TAAAGCTCTA AATCGGAACC CTAAAGGGAG
5941 CCCCCGATTT AGAGCTTGAC GGGGAAAGCC GGCGAACGTG GCGAGAAAGG AAGGGAAGAA
6001 AGCGAAAGGA GCGGGCGCTA GGGCGCTGGC AAGTGTAGCG GTCACGCTGC GCGTAACCAC
6061 CACACCCGCC GCGCTTAATG CGCCGCTACA GGGCGCGTCC ATTCGCCATT CAGGCTGCGC
6121 AACTGTTGGG AAGGGCGATC GGTGCGGGCC TCTTCGCTAT TACGCCAGCT GGCGAAAGGG
6181 GGATGTGCTG CAAGGCGATT AAGTTGGGTA ACGCCAGGGT TTTCCCAGTC ACGACGTTGT
6241 AAAACGACGG CCAGTGAATT GTAATACGAC TCACTATA

pA.S

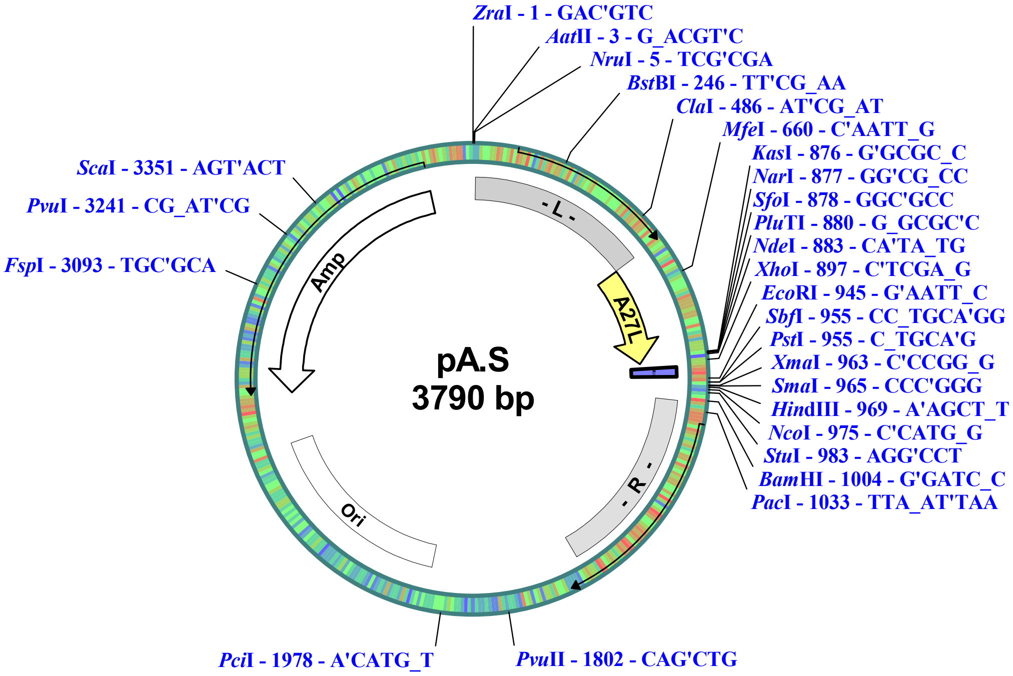


```
   1 CGTCGCGAGG TTAGAATCAT ACCATCGGTT CGATATGACA AAAGTAGATG TTGAACTATT
  61 TATTAAAGCA TTGTCTGATG CGTGTAAAAA AATTTTGTCA GCTTCTAATA GATTATAAAT
 121 GAACTCTCTA TCAATTTTTT TTATTGTGGT AGCGACGGCT GCGGTGTGTT TACTTTTTAT
 181 CCAGGGTTAC TCAATATATG AAAATTATGG CAATATTAAG GAATTTAATG CTACTCATGC
 241 AGCATTCGAA TATTCAAAAT CTATAGGTGG AACACCGGCA TTAGATAGGA GAGTTCAAGA
 301 TGTCAACGAC ACAATTTCTG ATGTAAAGCA AAAGTGGAGA TGTGTGGTTT ATCCAGGAAA
 361 CGGTTTTGTA TCCGCTTCCA TATTTGGATT TCAGGCAGAA GTTGGACCCA ATAATACTAG
 421 ATCCATTAGA AAATTTAACA CGATGCAACA ATGTATAGAC TTTACATTTT CTGATGTTAT
 481 TAACATCGAT ATTTATAATC CATGTGTTGT ACCAAATATA AATAACGCAG AGTGTCAGTT
 541 TCTAAAATCT GTACTTTAAA TGGACGGAAC TCTTTTCCCC GGAGATGACG ATCTTGCAAT
 601 TCCAGCAACT GAATTTTTTT CTACAAAGGC TGCTAAAAAG CCAGAGGCTA AACGCGAAGC
 661 AATTGTTAAA GCCGATGAAG ACGACAATGA GGAAACTCTC AAACAACGGC TAACTAATTT
 721 GGAAAAAAAG ATTACTAATG TAACAACAAA GTTTGAACAA ATAGAAAAGT GTTGTAAACG
 781 CAACGATGAA GTTCTATTTA GGTTGGAAAA TCACGCTGAA ACTCTAAGAG CGGCTATGAT
 841 ATCTCTGGCT AAAAAGATTG ATGTTCAGAC TGGACGGCGC CCATATGAGT AACTAACTCG
 901 AGAAAAATTG AAATTTTATT TTTTTTTTTT GGAATATAAA TAAGGAATTC CTGCAGGCTA
 961 GCCCCGGGAA GCTTCCATGG AGGCCTAAAT AAATAATTTT TATGGATCCG AGTAACTTAA
1021 CTCTTTTGTT AATTAAAAGT ATATTCAAAA AATGAGTTAT ATAAATGGCG AACATTATAA
1081 ATTTATGGAA CGGAATTGTA CCAACGGTTC AAGATGTTAA TGTTGCGAGC ATTACTGCGT
1141 TTAAATCTAT GATAGATGAA ACATGGGATA AAAAAATCGA AGCAAATACA TGCATCAGTA
1201 GAAAACATAG AAACATTATT CACGAAGTTA TTAGGGACTT TATGAAAGCC TATCCTAAAA
1261 TGGATGAGAA TAAAAAATCT CCATTAGGAG CCCCAATGCA ATGGCTAACA CAATATTATA
1321 TTTTAAAGAA TGAATATCAT AAGACCATGC TAGCGTATGA TAATGGATCA TTGAATACAA
1381 AATTTAAAAC GTTAAACATT TATATGATTA CTAACGTTGG TCAATATATT TTATATATAG
1441 TATTTTGTAT AATATCTGGT AAGAATCACG ATGGTACTCC TTATATATAC GATTCTGAGA
1501 TAACGAGCAA TGATAAAAAT TTTATTAATG AGCGTATCAA GTATGCATGT AAGCAAATAT
1561 TACACGGTCA ATTAACGATA TCGGATGCCG GGACCGACGA GTGCAGAGGC GTGCAAGCGA
1621 GCTTGGCGTA ATCATGGTCA TAGCTGTTTC CTGTGTGAAA TTGTTATCCG CTCACAATTC
1681 CACACAACAT ACGAGCCGGA AGCATAAAGT GTAAAGCCTG GGGTGCCTAA TGAGTGAGCT
1741 AACTCACATT AATTGCGTTG CGCTCACTGC CCGCTTTCCA GTCGGGAAAC CTGTCGTGCC
1801 AGCTGCATTA ATGAATCGGC CAACGCGCGG GGAGAGGCGG TTTGCGTATT GGGCGCTCTT
1861 CCGCTTCCTC GCTCACTGAC TCGCTGCGCT CGGTCGTTCG GCTGCGGCGA GCGGTATCAG
1921 CTCACTCAAA GGCGGTAATA CGGTTATCCA CAGAATCAGG GGATAACGCA GGAAAGAACA
1981 TGTGAGCAAA AGGCCAGCAA AAGGCCAGGA ACCGTAAAAA GGCCGCGTTG CTGGCGTTTT
2041 TCCATAGGCT CCGCCCCCCT GACGAGCATC ACAAAAATCG ACGCTCAAGT CAGAGGTGGC
2101 GAAACCCGAC AGGACTATAA AGATACCAGG CGTTTCCCCC TGGAAGCTCC CTCGTGCGCT
2161 CTCCTGTTCC GACCCTGCCG CTTACCGGAT ACCTGTCCGC CTTTCTCCCT TCGGGAAGCG
```

2221 TGGCGCTTTC TCATAGCTCA CGCTGTAGGT ATCTCAGTTC GGTGTAGGTC GTTCGCTCCA
2281 AGCTGGGCTG TGTGCACGAA CCCCCCGTTC AGCCCGACCG CTGCGCCTTA TCCGGTAACT
2341 ATCGTCTTGA GTCCAACCCG GTAAGACACG ACTTATCGCC ACTGGCAGCA GCCACTGGTA
2401 ACAGGATTAG CAGAGCGAGG TATGTAGGCG GTGCTACAGA GTTCTTGAAG TGGTGGCCTA
2461 ACTACGGCTA CACTAGAAGA ACAGTATTTG GTATCTGCGC TCTGCTGAAG CCAGTTACCT
2521 TCGGAAAAAG AGTTGGTAGC TCTTGATCCG GCAAACAAAC CACCGCTGGT AGCGGTGGTT
2581 TTTTTGTTTG CAAGCAGCAG ATTACGCGCA GAAAAAAAGG ATCTCAAGAA GATCCTTTGA
2641 TCTTTTCTAC GGGGTCTGAC GCTCAGTGGA ACGAAAACTC ACGTTAAGGG ATTTTGGTCA
2701 TGAGATTATC AAAAAGGATC TTCACCTAGA TCCTTTTAAA TTAAAAATGA AGTTTTAAAT
2761 CAATCTAAAG TATATATGAG TAAACTTGGT CTGACAGTTA CCAATGCTTA ATCAGTGAGG
2821 CACCTATCTC AGCGATCTGT CTATTTCGTT CATCCATAGT TGCCTGACTC CCCGTCGTGT
2881 AGATAACTAC GATACGGGAG GGCTTACCAT CTGGCCCCAG TGCTGCAATG ATACCGCGAG
2941 ACCCACGCTC ACCGGCTCCA GATTTATCAG CAATAAACCA GCCAGCCGGA AGGGCCGAGC
3001 GCAGAAGTGG TCCTGCAACT TTATCCGCCT CCATCCAGTC TATTAATTGT TGCCGGGAAG
3061 CTAGAGTAAG TAGTTCGCCA GTTAATAGTT TGCGCAACGT TGTTGCCATT GCTACAGGCA
3121 TCGTGGTGTC ACGCTCGTCG TTTGGTATGG CTTCATTCAG CTCCGGTTCC CAACGATCAA
3181 GGCGAGTTAC ATGATCCCCC ATGTTGTGCA AAAAAGCGGT TAGCTCCTTC GGTCCTCCGA
3241 TCGTTGTCAG AAGTAAGTTG GCCGCAGTGT TATCACTCAT GGTTATGGCA GCACTGCATA
3301 ATTCTCTTAC TGTCATGCCA TCCGTAAGAT GCTTTTCTGT GACTGGTGAG TACTCAACCA
3361 AGTCATTCTG AGAATAGTGT ATGCGGCGAC CGAGTTGCTC TTGCCCGGCG TCAATACGGG
3421 ATAATACCGC GCCACATAGC AGAACTTTAA AAGTGCTCAT CATTGGAAAA CGTTCTTCGG
3481 GGCGAAAACT CTCAAGGATC TTACCGCTGT TGAGATCCAG TTCGATGTAA CCCACTCGTG
3541 CACCCAACTG ATCTTCAGCA TCTTTTACTT TCACCAGCGT TTCTGGGTGA GCAAAAACAG
3601 GAAGGCAAAA TGCCGCAAAA AAGGGAATAA GGGCGACACG GAAATGTTGA ATACTCATAC
3661 TCTTCCTTTT TCAATATTAT TGAAGCATTT ATCAGGGTTA TTGTCTCATG AGCGGATACA
3721 TATTTGAATG TATTTAGAAA AATAAACAAA TAGGGGTTCC GCGCACATTT CCCCGAAAAG
3781 TGCCACCTGA
//

pA.S-GFP (pA.S-TagGFP2)

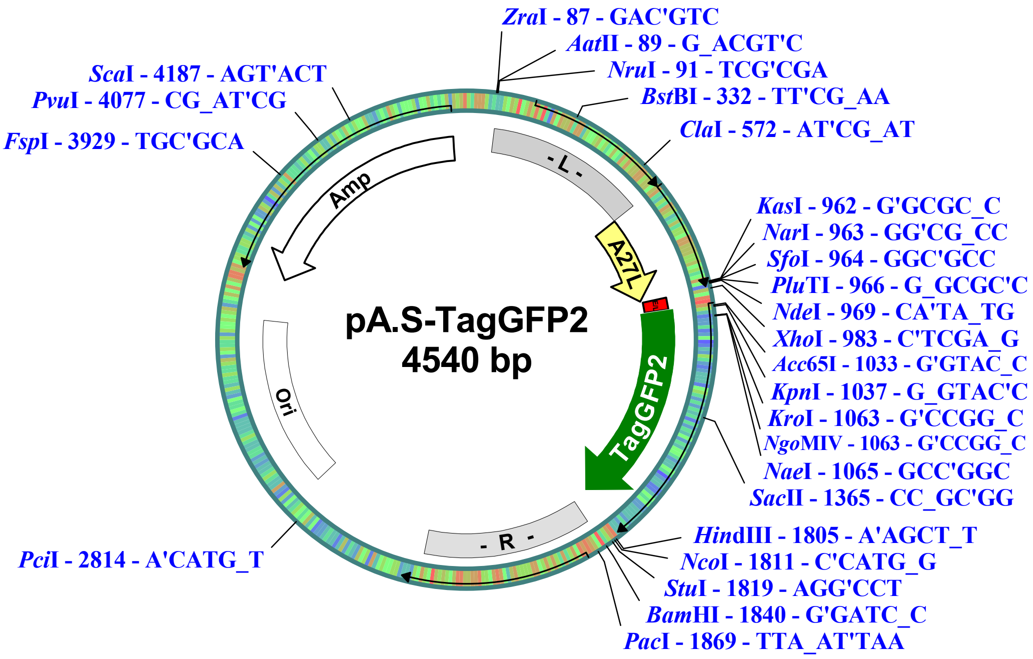


```
   1 CTCATGAGCG GATACATATT TGAATGTATT TAGAAAAATA AACAAATAGG GGTTCCGCGC
  61 ACATTTCCCC GAAAAGTGCC ACCTGACGTC GCGAGGTTAG AATCATACCA TCGGTTCGAT
 121 ATGACAAAAG TAGATGTTGA ACTATTTTATT AAAGCATTGT CTGATGCGTG TAAAAAAATT
 181 TTGTCAGCTT CTAATAGATT ATAAATGAAC TCTCTATCAA TTTTTTTTAT TGTGGTAGCG
 241 ACGGCTGCGG TGTGTTTACT TTTTATCCAG GGTTACTCAA TATATGAAAA TTATGGCAAT
 301 ATTAAGGAAT TTAATGCTAC TCATGCAGCA TTCGAATATT CAAAATCTAT AGGTGGAACA
 361 CCGGCATTAG ATAGGAGAGT TCAAGATGTC AACGACACAA TTTCTGATGT AAAGCAAAAG
 421 TGGAGATGTG TGGTTTATCC AGGAAACGGT TTTGTATCCG CTTCCATATT TGGATTTCAG
 481 GCAGAAGTTG GACCCAATAA TACTAGATCC ATTAGAAAAT TTAACACGAT GCAACAATGT
 541 ATAGACTTTA CATTTTCTGA TGTTATTAAC ATCGATATTT ATAATCCATG TGTTGTACCA
 601 AATATAAATA ACGCAGAGTG TCAGTTTCTA AAATCTGTAC TTTAAATGGA CGGAACTCTT
 661 TTCCCCGGAG ATGACGATCT TGCAATTCCA GCAACTGAAT TTTTTTCTAC AAAGGCTGCT
 721 AAAAAGCCAG AGGCTAAACG CGAAGCAATT GTTAAAGCCG ATGAAGACGA CAATGAGGAA
 781 ACTCTCAAAC AACGGCTAAC TAATTTGGAA AAAAAGATTA CTAATGTAAC AACAAAGTTT
 841 GAACAAATAG AAAAGTGTTG TAAACGCAAC GATGAAGTTC TATTTAGGTT GGAAAATCAC
 901 GCTGAAACTC TAAGAGCGGC TATGATATCT CTGGCTAAAA AGATTGATGT TCAGACTGGA
 961 CGGCGCCCAT ATGAGTAACT AACTCGAGAA AAATTGAAAT TTTATTTTTT TTTTTTTGGA
1021 ATATAAATGA GCGGTACCGG AAGCGGGGGC GAGGAGCTGT TCGCCGGCAT CGTGCCCGTG
1081 CTGATCGAGC TGGACGGCGA CGTGCACGGC CACAAGTTCA GCGTGCGCGG CGAGGGCGAG
1141 GGCGACGCCG ACTACGGCAA GCTGGAGATC AAGTTCATCT GCACCACCGG CAAGCTGCCC
1201 GTGCCCTGGC CCACCCTGGT GACCACCCTC TGCTACGGCA TCCAGTGCTT CGCCCGCTAC
1261 CCCGAGCACA TGAAGATGAA CGACTTCTTC AAGAGCGCCA TGCCCGAGGG CTACATCCAG
1321 GAGCGCACCA TCCAGTTCCA GGACGACGGC AAGTACAAGA CCCGCGGCGA GGTGAAGTTC
1381 GAGGGCGACA CCCTGGTGAA CCGCATCGAG CTGAAGGGCA AGGACTTCAA GGAGGACGGC
1441 AACATCCTGG GCCACAAGCT GGAGTACAGC TTCAACAGCC ACAACGTGTA CATCCGCCCC
1501 GACAAGGCCA ACAACGGCCT GGAGGCTAAC TTCAAGACCC GCCACAACAT CGAGGGCGGC
1561 GGCGTGCAGC TGGCCGACCA CTACCAGACC AACGTGCCCC TGGGCGACGG CCCCGTGCTG
1621 ATCCCCATCA ACCACTACCT GAGCACTCAG ACCAAGATCA GCAAGGACCG CAACGAGGCC
1681 CGCGACCACA TGGTGCTCCT GGAGTCCTTC AGCGCCTGCT GCCACACCCA CGGCATGGAC
1741 GAGCTGTACA GGTCCGGAGG TGGAGGTTCC GGAGCTAGCC AATTGTTATA ATTTTTCTTT
1801 TTCTAAGCTT CCATGGAGGC CTAAATAAAT AATTTTTATG GATCCGAGTA ACTTAACTCT
1861 TTTGTTAATT AAAAGTATAT TCAAAAAATG AGTTATATAA ATGGCGAACA TTATAAATTT
1921 ATGGAACGGA ATTGTACCAA CGGTTCAAGA TGTTAATGTT GCGAGCATTA CTGCGTTTAA
1981 ATCTATGATA GATGAAACAT GGGATAAAAA AATCGAAGCA AATACATGCA TCAGTAGAAA
2041 ACATAGAAAC ATTATTCACG AAGTTATTAG GGACTTTATG AAAGCCTATC CTAAAATGGA
2101 TGAGAATAAA AAATCTCCAT TAGGAGCCCC AATGCAATGG CTAACACAAT ATTATATTTT
2161 AAAGAATGAA TATCATAAGA CCATGCTAGC GTATGATAAT GGATCATTGA ATACAAAATT
```

2221 TAAAACGTTA AACATTTATA TGATTACTAA CGTTGGTCAA TATATTTTAT ATATAGTATT
2281 TTGTATAATA TCTGGTAAGA ATCACGATGG TACTCCTTAT ATATACGATT CTGAGATAAC
2341 GAGCAATGAT AAAAATTTTA TTAATGAGCG TATCAAGTAT GCATGTAAGC AAATATTACA
2401 CGGTCAATTA ACGATATCGG ATGCCGGGAC CGACGAGTGC AGAGGCGTGC AAGCGAGCTT
2461 GGCGTAATCA TGGTCATAGC TGTTTCCTGT GTGAAATTGT TATCCGCTCA CAATTCCACA
2521 CAACATACGA GCCGGAAGCA TAAAGTGTAA AGCCTGGGGT GCCTAATGAG TGAGCTAACT
2581 CACATTAATT GCGTTGCGCT CACTGCCCGC TTTCCAGTCG GGAAACCTGT CGTGCCAGCT
2641 GCATTAATGA ATCGGCCAAC GCGCGGGGAG AGGCGGTTTG CGTATTGGGC GCTCTTCCGC
2701 TTCCTCGCTC ACTGACTCGC TGCGCTCGGT CGTTCGGCTG CGGCGAGCGG TATCAGCTCA
2761 CTCAAAGGCG GTAATACGGT TATCCACAGA ATCAGGGGAT AACGCAGGAA AGAACATGTG
2821 AGCAAAAGGC CAGCAAAAGG CCAGGAACCG TAAAAAGGCC GCGTTGCTGG CGTTTTTCCA
2881 TAGGCTCCGC CCCCCTGACG AGCATCACAA AAATCGACGC TCAAGTCAGA GGTGGCGAAA
2941 CCCGACAGGA CTATAAAGAT ACCAGGCGTT TCCCCCTGGA AGCTCCCTCG TGCGCTCTCC
3001 TGTTCCGACC CTGCCGCTTA CCGGATACCT GTCCGCCTTT CTCCCTTCGG GAAGCGTGGC
3061 GCTTTCTCAT AGCTCACGCT GTAGGTATCT CAGTTCGGTG TAGGTCGTTC GCTCCAAGCT
3121 GGGCTGTGTG CACGAACCCC CCGTTCAGCC CGACCGCTGC GCCTTATCCG GTAACTATCG
3181 TCTTGAGTCC AACCCGGTAA GACACGACTT ATCGCCACTG GCAGCAGCCA CTGGTAACAG
3241 GATTAGCAGA GCGAGGTATG TAGGCGGTGC TACAGAGTTC TTGAAGTGGT GGCCTAACTA
3301 CGGCTACACT AGAAGAACAG TATTTGGTAT CTGCGCTCTG CTGAAGCCAG TTACCTTCGG
3361 AAAAAGAGTT GGTAGCTCTT GATCCGGCAA ACAAACCACC GCTGGTAGCG GTGGTTTTTT
3421 TGTTTGCAAG CAGCAGATTA CGCGCAGAAA AAAAGGATCT CAAGAAGATC CTTTGATCTT
3481 TTCTACGGGG TCTGACGCTC AGTGGAACGA AAACTCACGT TAAGGGATTT TGGTCATGAG
3541 ATTATCAAAA AGGATCTTCA CCTAGATCCT TTTAAATTAA AAATGAAGTT TTAAATCAAT
3601 CTAAAGTATA TATGAGTAAA CTTGGTCTGA CAGTTACCAA TGCTTAATCA GTGAGGCACC
3661 TATCTCAGCG ATCTGTCTAT TTCGTTCATC CATAGTTGCC TGACTCCCCG TCGTGTAGAT
3721 AACTACGATA CGGGAGGGCT TACCATCTGG CCCCAGTGCT GCAATGATAC CGCGAGACCC
3781 ACGCTCACCG GCTCCAGATT TATCAGCAAT AAACCAGCCA GCCGGAAGGG CCGAGCGCAG
3841 AAGTGGTCCT GCAACTTTAT CCGCCTCCAT CCAGTCTATT AATTGTTGCC GGGAAGCTAG
3901 AGTAAGTAGT TCGCCAGTTA ATAGTTTGCG CAACGTTGTT GCCATTGCTA CAGGCATCGT
3961 GGTGTCACGC TCGTCGTTTG GTATGGCTTC ATTCAGCTCC GGTTCCCAAC GATCAAGGCG
4021 AGTTACATGA TCCCCCATGT TGTGCAAAAA AGCGGTTAGC TCCTTCGGTC CTCCGATCGT
4081 TGTCAGAAGT AAGTTGGCCG CAGTGTTATC ACTCATGGTT ATGGCAGCAC TGCATAATTC
4141 TCTTACTGTC ATGCCATCCG TAAGATGCTT TTCTGTGACT GGTGAGTACT CAACCAAGTC
4201 ATTCTGAGAA TAGTGTATGC GGCGACCGAG TTGCTCTTGC CCGGCGTCAA TACGGGATAA
4261 TACCGCGCCA CATAGCAGAA CTTTAAAAGT GCTCATCATT GGAAAACGTT CTTCGGGGCG
4321 AAAACTCTCA AGGATCTTAC CGCTGTTGAG ATCCAGTTCG ATGTAACCCA CTCGTGCACC
4381 CAACTGATCT TCAGCATCTT TTACTTTCAC CAGCGTTTCT GGGTGAGCAA AAACAGGAAG
4441 GCAAAATGCC GCAAAAAAGG GAATAAGGGC GACACGGAAA TGTTGAATAC TCATACTCTT
4501 CCTTTTTCAA TATTATTGAA GCATTTATCA GGGTTATTGT
//
